# InDel Markers for Identifying Interspecific Hybrid Offspring of Apple and Pear

**DOI:** 10.3390/plants14050646

**Published:** 2025-02-20

**Authors:** Wenqi Fan, Baoan Wang, Yao Xiao, Jinfeng Huang, Dongmei Wang, Shengyuan Wang, Wei Li, Qiulei Zhang, Fuli Huang, Chenxi Shi, Tianzhong Li

**Affiliations:** 1 Laboratory of Fruit Cell and Molecular Breeding, China Agricultural University, Beijing 100193, China; fanwenqi639@163.com (W.F.); poanwang@163.com (B.W.); xiaoyao6703@163.com (Y.X.); wsy0515@yeah.net (S.W.); jason870613@126.com (W.L.); zqiul880807@163.com (Q.Z.); hfll18101217780@163.com (F.H.); shicx5@163.com (C.S.); 2 Liaoning Institute of Pomology, Yingkou 115009, China; huangfeng1002@163.com (J.H.); lgwdm@163.com (D.W.)

**Keywords:** distant hybridization, species identification, whole-genome resequencing, InDel markers

## Abstract

Distant hybridization, such as between apple (*Malus domestica* Borkh.) and pear (*Pyrus communis* L.), can introduce unique genetic traits that aren’t possible through traditional hybridization methods. However, these hybrids often display maternal traits, making it difficult to visually identify hybrid offspring in the early stages. Thus, identifying these hybrids has been a longstanding challenge. To address this challenge, this study performed whole-genome resequencing of apple ‘Golden Delicious’ and pear ‘Yan Zhuang’, along with 62 of their hybrid offspring, to develop effective molecular markers. Genomic variation analysis revealed significant genetic diversity between apple and pear. By selecting markers based on notable parental differences and high polymorphism among offspring, 51 effective markers were identified from 340 apple-specific InDel markers. These markers could reliably identify the 62 offspring as distant hybrids with genetic material from both apple and pear. Among them, InDel1-7, InDel2-3, InDel3-1, and InDel4-3 proved to be the most efficient, achieving a 100% identification rate for hybrid offspring when used in combination in the ‘Golden Delicious’ × ‘Yan Zhuang’ population. Furthermore, marker universality tests showed that 88.2%, 76.5%, and 70.6% of the 51 markers were transferable to the ‘Golden Delicious’ × ‘Jin Zhui’, ‘Fuji’ × ‘Yan Zhuang’, and ‘Fuji’ × ‘Jin Zhui’ hybrid populations, respectively. The identification efficiencies in these three validation populations were 82.6%, 80.3%, and 85.0%, with the four highly efficient markers exceeding a 70% identification rate. This study developed an efficient molecular marker system for identifying apple and pear distant hybrid offspring based on InDel variation, providing a valuable tool for breeding new varieties of Rosaceae fruit trees.

## 1. Introduction

Distant hybridization can break species barriers, integrating biological traits from two different species, genera, or higher taxonomic groups into a single entity, thereby creating novel genetic variations and fertile progenies. The introduction of alien genes through distant hybridization has become an important means of expanding genetic variation in plants [1,2,3,4], and this approach has been widely applied in the breeding of crops such as *Oryza sativa* L. (rice), *Zea mays* L. (corn), *Glycine max* L. (soybean), *Brassica napus* L. (rapeseed), *Triticum aestivum* L. (wheat), and *Gossypium* spp. (cotton). Compared to annual crops, perennial fruit species face greater challenges in achieving successful distant hybridization due to their complex genetic backgrounds and extended juvenile phases [5]. Nevertheless, notable successes have been documented within the Rosaceae family, including intergeneric hybrids between *Prunus salicina* Lindl. (plum) and *Prunus armeniaca* L. (apricot) [6], *Sinocalycanthus chinensis* and *Calycanthus floridus* [7], *Pyrus pyrifolia* (pear) and *Eriobotrya japonica* (loquat) [8], and *Amygdalus persica* L. (peach) and *Prunus armeniaca* L. (apricot) [9], as well as distant hybrids between *Malus domestica* (apple) and *Pyrus* spp. (pear) [10,11,12,13,14,15]. A critical observation from historical distant hybridization efforts reveals that hybrid progenies of fruit crops frequently exhibit phenotypic traits highly analogous to their maternal parents, rendering conventional morphological identification unreliable. This underscores the imperative to develop molecular authentication methodologies for accurate hybrid verification, which would significantly accelerate distant hybridization breeding programs and shorten the breeding cycle.

While methodologies developed over past decades—including morphological observation, cytological analysis, and isozyme markers—have laid the foundation for authenticating intergeneric hybrids between *Malus domestica* (apple) and *Pyrus* spp. (pear) [13,16], DNA-based molecular markers such as restriction fragment length polymorphism (RFLP), amplified fragment length polymorphism (AFLP), random amplified polymorphic DNA (RAPD), and simple sequence repeat (SSR) have been extensively employed in hybrid germplasm identification across plant species [17,18,19,20,21]. The third-generation DNA markers represented by SNPs and InDels, which emerged with the advancement of resequencing technology [22], have gained widespread application in plant genetic research due to their codominant nature, high-density distribution, and technical accessibility. Unlike SNP markers that require validation through restriction enzyme digestion [23], InDel markers are developed based on nucleotide insertions or deletions in homologous sequences among different genotypes [24], exhibiting combined characteristics of SSRs and SNPs. Currently, InDel markers have been widely utilized in various aspects of crop research, including the construction of high-density molecular marker genetic maps, gene mapping, genetic diversity analysis, and hybrid progeny identification. Liu et al. (2013) developed InDel markers based on whole-genome resequencing of Brassica rapa, and constructed a high-density genetic linkage map, providing a valuable resource for genetic analysis and fine mapping of molecular traits [25]. Li et al. (2015) developed InDel markers based on *Capsicum annuum* and utilized these markers to construct a genetic linkage map [26]. In recent years, InDel markers have begun to be developed and applied in fruit tree breeding programs. Through whole-genome resequencing (WGR) combined with bulked segregant analysis (BSA), SNP and InDel markers closely linked to gene loci associated with apple resistance to anthracnose and leaf blight were identified [27]. Marti et al. (2018) constructed a physical map of Japanese plum (*Prunus salicina*) based on SNPs and InDels obtained from genome sequencing, providing a foundation for molecular marker-assisted breeding [28]. However, in the context of distant hybridization between apple and pear, the development of molecular markers for early identification of hybrid progeny remains in the exploratory stage.

Our team has previously conducted distant hybridization breeding experiments between apple and pear, resulting in the establishment of four distinct hybrid populations. To accurately identify the progeny derived from these distant crosses, this study developed an InDel molecular marker based on resequencing data from the hybrid populations. This marker exhibits broad applicability across multiple apple–pear hybrid populations with diverse genetic backgrounds. The molecular marker tool developed herein significantly shortens the breeding cycle of apple–pear hybrids, enhances the accuracy of hybrid progeny identification, and accelerates the innovation of new germplasm in apple–pear hybridization. Furthermore, this study provides a theoretical reference for distant hybridization breeding in horticultural crops.

## 2. Results

### 2.1. Construction of a Distant Hybridization Population Between Apple and Pear

**Figure 1:**
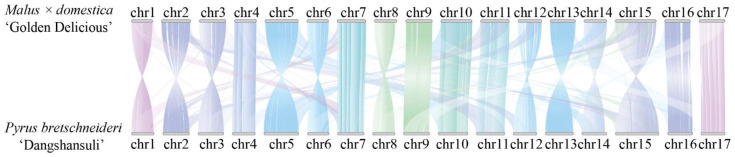
Genomic collinearity analysis of apple and pear.

Synteny analysis revealed a high level of genomic collinearity between the chromosomes of apple and pear, suggesting a close evolutionary relationship between the two species (Figure 1). This forms the basis for the successful distant hybridization between apple and pear. To obtain their distant hybrid offspring, this study conducted distant hybridization using the pear varieties ‘Yan Zhuang’ and ‘Jin Zhui’ as male parents, and the apple varieties ‘Golden Delicious’ and ‘Fuji’ as female parents, resulting in four intergeneric hybrid offspring populations: ‘Golden Delicious’ × ‘Yan Zhuang’, ‘Golden Delicious’ × ‘Jin Zhui’, ‘Fuji’ × ‘Yan Zhuang’, and ‘Fuji’ × ‘Jin Zhui’, which included 62, 46, 76, and 40 offspring, respectively. It was observed that the fruit set rate of distant hybridization was low, ranging from 20.6% to 26.4%, and the germination rate of offspring seeds was below 50% (Table 1).

### 2.2. Resequencing of Distant Hybridization Progeny and Genome-Wide Genetic Variations

**Figure 2:**
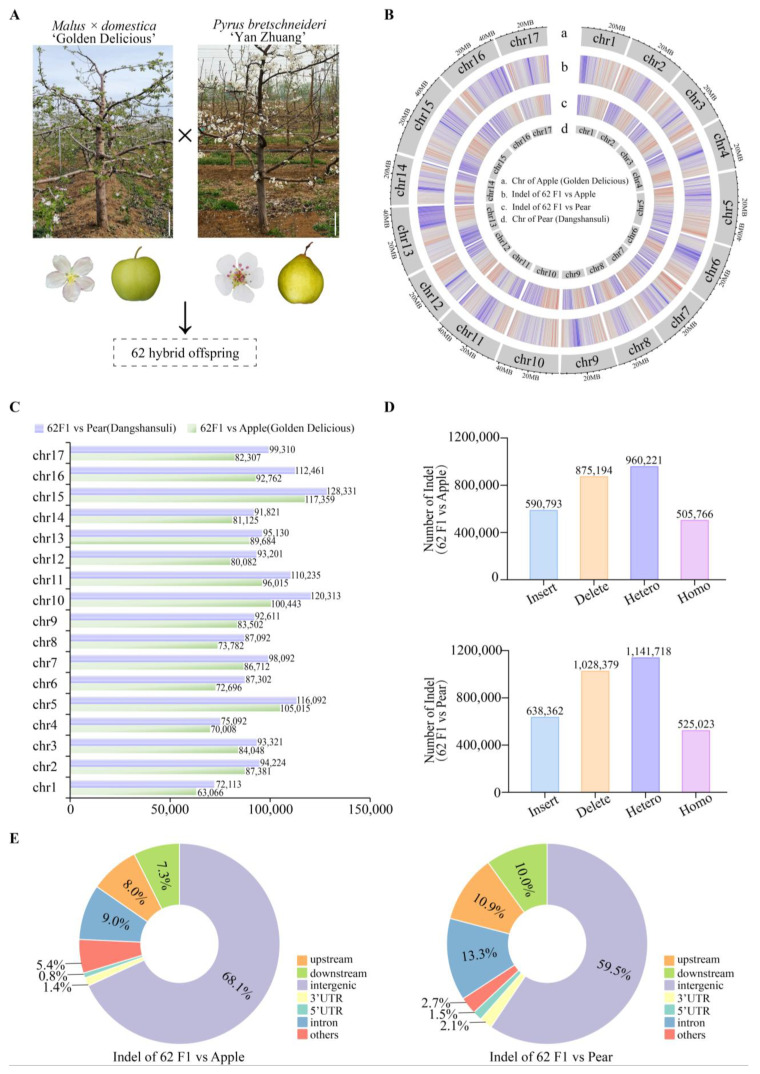
Sequencing population construction and whole-genome genetic variation analysis. (**A**) Total of 62 hybrid offspring were generated from the cross between the apple cultivar ‘Golden Delicious’ and the pear cultivar ‘Yan Zhuang’. Bar, 0.5 m. (**B**) Genomic variation circle plot of resequencing offspring. a: chromosome of apple reference genome ‘Golden Delicious’, b: InDel of 62F1 vs. apple, c: InDel of 62F1 vs. pear, d: chromosome of pear reference genome ‘Dangshansuli’. The red line indicates higher InDel density, while the blue line indicates lower InDel density. (**C**) The distribution of InDels on the chromosomes. (**D**) Type statistics of InDel variations. (**E**) Distribution of InDels in different gene regions.

In this study, we conducted whole-genome resequencing of the female parent ‘Golden Delicious’, the male parent ‘Yan Zhuang’, and their 62 hybrid offspring (Figure 2A). The published genomes of *Malus* × *domestica* ‘Golden Delicious’ and *Pyrus brestschneideri* ‘Dangshansuli’ were used as the reference genomes for the female and male parents, respectively. Analysis of the parental resequencing data revealed 84,249,690 and 75,193,854 paired-end clean reads for the maternal and paternal parents, respectively, with an average coverage depth of approximately 20.9-fold and 19.7-fold relative to their reference genomes. The overall mapping ratios to their respective reference genomes were 98.8% and 95.9%. Furthermore, the cover ratios of sequencing reads mapped to the reference genome were 94.1% and 91.2%, and 38.2% and 37.1% of high GC-content regions were covered. Finally, the analysis of the resequencing data for the 62 hybrid offspring, using the aforementioned two genomes as references, also indicated high sequencing quality and reliable data (Table 2).

InDel variation analysis, by aligning the 62 hybrid offspring with the apple and pear reference genomes (Figure 2B), discovered a total of 1,465,987 and 1,666,741 InDels across 17 chromosomes (Figure 2C). Insertion-type InDels accounted for 590,793 and 638,362, while deletion-type InDels accounted for 875,194 and 1,028,379. Heterozygous InDel sites were 960,221 and 1,141,718, and homozygous InDel sites were 505,766 and 525,023, respectively (Figure 2D). The positional distribution statistics indicate that InDels were distributed in intergenic regions, upstream of genes, downstream of genes, intronic regions, 3′ UTR regions, and 5′ UTR regions, with the majority located in intergenic regions, accounting for 68.1% and 59.5%, respectively (Figure 2E).

### 2.3. Development of Identification Markers for Distant Hybridization Progeny

**Figure 3:**
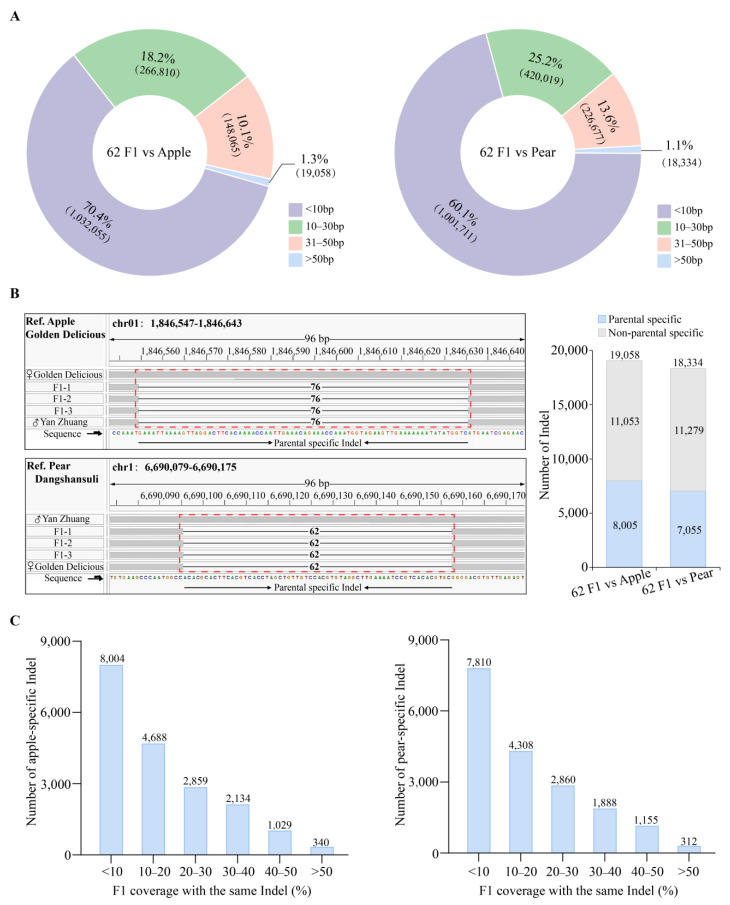
Development process of identification markers for offspring from distant hybridization. (**A**) InDel length classification of 62F1 vs. apple (left) and 62F1 vs. pear (right). (**B**) IGV visualization for identifying parental-specific InDels, as shown in the figure, a 76 bp apple-specific InDel and a 62 bp pear-specific InDel (left) and the corresponding parental-specific InDel statistical data (right). (**C**) F1 coverage with the same InDel of apple-specific InDel (left) and pear-specific InDel (right) in offspring.

To effectively identify the hybrid offspring, we initially quantified the lengths of the InDels and found that 1.3% (19,058) and 1.1% (18,334) of the InDels were greater than 50 bp in length (Figure 3A), which are suitable for PCR validation. Subsequently, by aligning the resequencing data of the hybrid offspring with the parental genomes (apple and pear), we identified InDel markers that exhibited differences between the parental resequencing data (Figure 3B), yielding 8005 apple-specific InDels and 7055 pear-specific InDels (Figure 3B). Analysis of the coverage of InDel markers in the offspring revealed that 340 apple-specific InDels (present in the apple mother but absent in the offspring) and 312 pear-specific InDels (present in the pear father but absent in the offspring) covered more than 50% of the offspring individuals (Figure 3C). Finally, considering the phenotype of the offspring tended towards apple, we aimed to identify hybrid offspring containing pear genetic material as distant hybrid varieties. Therefore, apple-specific InDels were selected for subsequent marker development.

**Figure 4:**
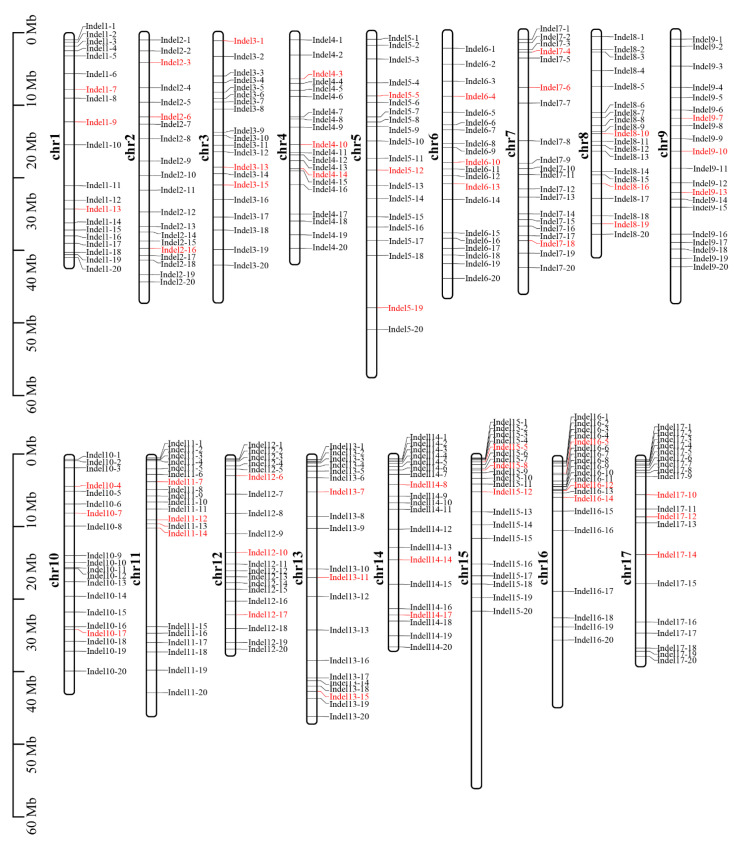
Distribution of 340 InDel markers on each chromosome. InDel marker names are listed to the right of the chromosomes. The ruler label to the left of chromosomes represents the physical distance. The red markers indicate 51 valid InDel markers.

**Figure 5:**
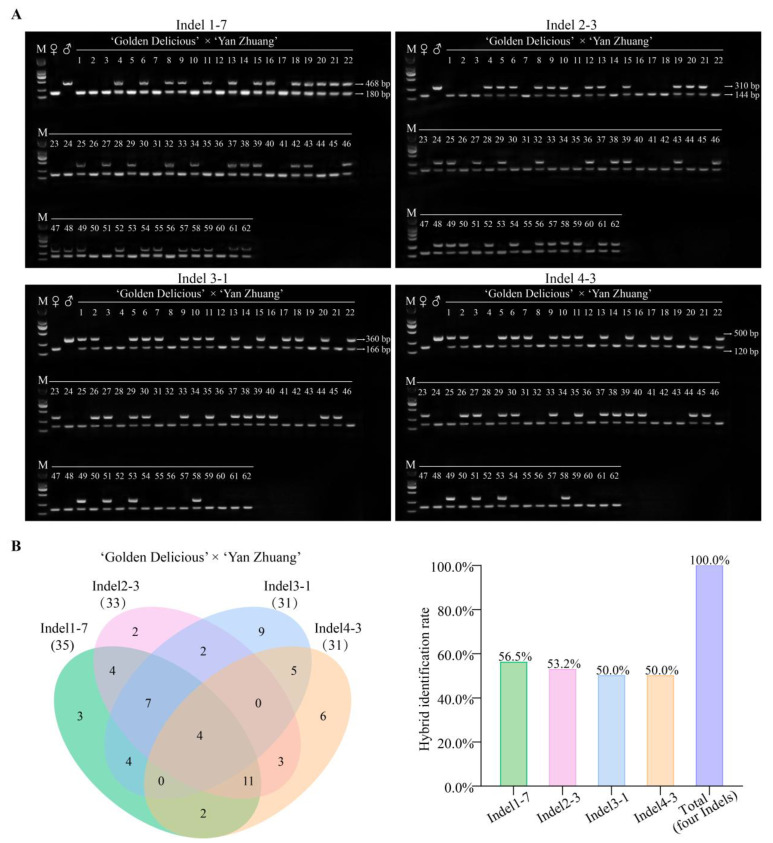
PCR validation and analysis of InDel markers. (**A**) In the 62 F1 progeny of the cross between ‘Golden Delicious’ × ‘Yan Zhuang’, PCR amplification of InDel markers InDel1-7, InDel2-3, InDel3-1, and InDel4-3 revealed that all of 62 individuals exhibited bands consistent with both parents, while the remaining offspring exhibited a single band matching the maternal parent. M, DNA Marker DL 2000. maternal parent, ‘Golden Delicious’. paternal parent, ‘Yan Zhuang’. (**B**) InDel1-7, InDel2-3, InDel3-1, and InDel4-3 markers identified a total of 35 (56.5%), 33 (53.2%), 31 (50.0%), and 31 (50.0%) true hybrids, respectively, summing up to 62 individuals (100.0%).

The 340 apple-specific InDels were evenly distributed across all 17 chromosomes, demonstrating the comprehensiveness of the markers (Figure 4). PCR primers were designed targeting these InDels (Table S1). Polymorphism identification of parental PCR markers revealed that 284 pairs of primers successfully amplified in both parents, with 128 pairs of primers producing polymorphic amplification, which could be further utilized for hybrid offspring identification, achieving a marker development efficiency of 45.1%. The offspring marker polymorphism PCR identification ultimately confirmed the effectiveness of 51 pairs of primers, which were able to authenticate all 62 offspring as true hybrids. Among these, InDel1-7, InDel2-3, InDel3-1, and InDel4-3 had the highest identification efficiency, identifying 35, 33, 31, and 31 true hybrids, respectively, with the combined use of these four primer pairs achieving a 100% identification rate (Figure 5).

### 2.4. Universality of Markers

**Figure 6:**
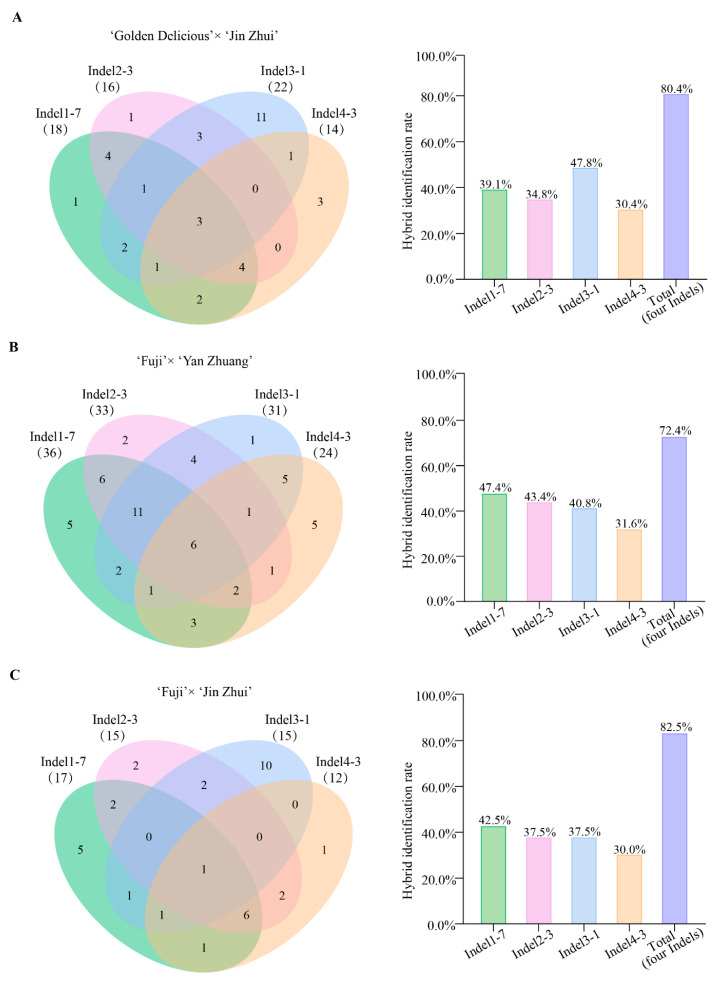
Validation information of InDel markers in three populations. (**A**) In the 46 F1 progeny of the ‘Golden Delicious’ × ‘Jin Zhui’ population, the number of individuals identified by four InDel markers (left) and the hybrid identification rate of the markers (right). (**B**) In the 76 F1 progeny of the ‘Fuji’ × ‘Yan Zhuang’ population, the number of individuals identified by four InDel markers (left) and the hybrid identification rate of the markers (right). (**C**) In the 40 F1 progeny of the ‘Fuji’ × ‘Jin Zhui’ population, the number of individuals identified by four InDel markers (left) and the hybrid identification rate of the markers (right).

To validate the universality of the markers, we further tested the identification efficacy of 51 InDel markers in three additional hybrid populations with different genetic backgrounds of apple and pear crosses. The results indicate that in the ‘Golden Delicious’ × ‘Jin Zhui’ population (46 offspring), ‘Fuji’ × ‘Yan Zhuang’ population (76 offspring), and ‘Fuji’ × ‘Jin Zhui’ population (40 offspring), there were 45, 39, and 36 pairs of primers exhibiting polymorphism, respectively, with a primer universality rate exceeding 70% (Table 3). Additionally, 38, 61, and 35 hybrid individuals were identified in each population, with hybridization rates of 82.6%, 80.3%, and 85.0%, respectively. Similarly, the four primer pairs InDel1-7, InDel2-3, InDel3-1, and InDel4-3 showed the highest identification efficiency, and their combined use achieved identification rates of 80.4%, 72.4%, and 82.5% in the ‘Golden Delicious’ × ‘Jin Zhui’, ‘Fuji’ × ‘Yan Zhuang’, and ‘Fuji’ × ‘Jin Zhui’ populations, respectively (Figure 6 and Figures S1–S3).

## 3. Discussion

Distant hybridization, as a critical approach to overcoming reproductive isolation between species and facilitating interspecific transfer of desirable genes, plays an indispensable role in fruit crop germplasm innovation [29,30,31,32,33]. Since 1952, continuous efforts have been devoted to apple (*Malus × domestica*) and pear (*Pyrus* spp.) distant hybridization breeding, with successful generation of hybrid progenies [10,11,12,13,14,15]. Notably, these hybrids frequently exhibit higher phenotypic resemblance to maternal parents [34], a characteristic that complicates early-stage hybrid identification. Conventional progeny identification in distant hybridization primarily relies on phenotypic observations, yet the efficacy of this method depends on the extent of trait segregation and variation in offspring while being susceptible to environmental influences. For instance, Toxopeus reported that while both pummelo (*Citrus maxima*) and *Citrus hystrix* possess unifoliolate leaves, approximately one-third of their hybrids displayed trifoliolate leaves [35]. Similarly, apple–pear hybrids demonstrate closer physiological trait similarities to maternal parents in leaf morphology, floral structures, and fruit characteristics [34]. Such phenotypic convergence toward maternal traits and limited variation significantly impedes hybrid identification. Recent technological advancements have provided potential solutions to this challenge. Chromosomal identification techniques, which distinguish hybrids from parental lines through ploidy analysis, have proven effective in hybrid verification for species such as grapes [36]. However, this approach is not applicable to homoploid species like apples and pears. Isozyme marker technology partially addressed hybrid identification in apple–pear crosses [37], yet its limitations, including low polymorphism detection and labor-intensive procedures, have restricted its widespread adoption.

With the advancement of modern molecular biology technologies, molecular markers have emerged as powerful tools for assessing genetic diversity and phylogenetic relationships in biological populations. Among these, insertion–deletion (InDel) markers have been widely applied due to their high polymorphism, ease of detection, and genetic stability [38,39,40,41]. Notable examples include their use in precise differentiation of *Citrus*, *Poncirus*, and *Fortunella* species [42], identification of hybrids between *Malus* (apple) and *Pyrus* (pear) [19], discrimination of *Actinidia arguta* cultivars [43], and characterization of distant hybrids between *Citrus grandis* (pomelo) and *Poncirus trifoliata* [44]. However, existing InDel markers in these studies were developed solely through parental genome comparisons without considering progeny genetic information, potentially limiting marker efficiency and coverage. The widespread adoption of resequencing technology has facilitated the design of high-performance molecular markers. In this study, we developed four highly efficient InDel markers by comprehensively integrating resequencing data from both parental lines and hybrid progeny populations, achieving 100% identification accuracy when markers were used combinatorially. Multi-population validation demonstrated exceptional universal applicability, with a universal applicability rate of 78.4% across diverse apple–pear hybrid progenies. Structural variations (e.g., DNA rearrangements) and chromosomal recombination events in different hybrid combinations were identified as potential genetic constraints affecting marker universality [45,46]. Similarly, Noda et al. developed 61 InDel markers through comparative analysis of *Citrus unshiu* genome and hybrid progeny resequencing data, which were further validated as effective genotyping tools for interspecific hybrids beyond *C. unshiu* [47]. Our findings not only provide a theoretical foundation for optimizing distant hybridization breeding systems between apple and pear but also establish a methodological framework transferable to interspecific breeding programs in other fruit crops.

## 4. Materials and Methods

### 4.1. Plant Materials

Using apple varieties ‘Golden Delicious’ and ‘Fuji’ planted at the base of the Liaoning Institute of Pomology as the maternal parents, and the pear varieties ‘Yan Zhuang’ and ‘Jin Zhui’ planted at the Shangzhuang Experimental Station of China Agricultural University in Beijing as the paternal parents, we carried out distant hybridization breeding. Through hybridization, we generated four distant hybrid populations: ‘Golden Delicious’ × ‘Yan Zhuang’ (62 offspring), ‘Golden Delicious’ × ‘Jin Zhui’ (46 offspring), ‘Fuji’ × ‘Yan Zhuang’ (76 offspring), and ‘Fuji’ × ‘Jin Zhui’ (40 offspring). Seeds were sown in the following spring for germination and seedling cultivation. Once the seedlings had developed multiple true leaves, their leaf samples were collected and immediately flash-frozen in liquid nitrogen. These samples were then stored at −80 °C for preservation, to be utilized in subsequent experiments.

### 4.2. Genomic DNA Library Preparation and Sequencing

Leaf samples were collected from the parental lines and hybrid offspring of ‘Golden Delicious’ × ‘Yan Zhuang’ population, with a total of 64 samples for genomic DNA extraction. DNA was extracted from the tissue using the CTAB method. The quality of the isolated genomic DNA was verified by using these two methods in combination: (1) DNA degradation and contamination were monitored on 1% agarose gels; (2) DNA concentration was measured by ND-2000 spectrophotometer (Thermo Fisher Scientific, Waltham, MA, USA). Only high-quality DNA samples with an OD260/280 ratio of 1.8 to 2.0 and an OD260/230 ratio of at least 2.0 were used to construct sequencing libraries.

For each sample, 0.5 μg of DNA was used as the starting material for library preparation. The sequencing library was constructed using the TruSeq Nano DNA HT Sample Prep Kit (Illumina, San Diego, CA, USA) according to the manufacturer’s protocol, with unique index codes incorporated into each sample. The workflow involved the following steps: genomic DNA was sonicated to achieve a fragment size of approximately 350 bp. Subsequently, the DNA fragments underwent end polishing, adenylation (A-tailing), and ligation with full-length adapters compatible with Illumina NovaSeq X Plus sequencing platforms, followed by PCR amplification. Post-amplification, the PCR products were purified using the AMPure XP system. Library size distribution was assessed with an Agilent 2100 Bioanalyzer (Agilent Technologies, Santa Clara, CA, USA), and library concentration was determined by real-time PCR to a final concentration of 3 nM. The paired-end DNA sequencing libraries were then sequenced on the Illumina NovaSeq X Plus platform (Illumina, USA) at Shanghai Majorbio Bio-pharm Technology Co., Ltd (Shanghai, China).

### 4.3. Bioinformatics Analysis

Gene annotation files and protein sequence files were prepared, and the Python (v3.11) of the MCScan program, JCVI, was utilized to perform sequence alignments between *Pyrus bretschneider* ‘Dangshansuli’ and *Malus × domestica* ‘Golden Delicious’. Similar sequences were identified, collinear regions were determined, and they were combined into syntenic blocks. The conservation of chromosomal regions between different species or individuals was assessed by examining whether the order and orientation of genes within the syntenic blocks were consistent across different species. Synteny analysis can aid in understanding evolutionary events such as genome duplication, recombination, and inversion.

### 4.4. Data Filtering, Sequence Alignment and InDel Calling

Low-quality raw reads, characterized by a mean Phred score below 20, as well as reads containing adapter contamination or an excessive number of unrecognizable nucleotides (N bases > 10), were either trimmed or discarded using the Fastp software (v0.23.0). The reads that remained after this quality control step were aligned to their respective references using the BWA-MEM algorithm [48] under default mapping parameters. Following the modified GATK Best Practices pipeline [49], the alignment BAM files were sorted using SAMtools, and PCR duplicates were marked with MarkDuplicated. After performing base quality recalibration, germline variant calling, which included InDels across all samples, was conducted using the Haplotyper and Gvcftyper programs in the Sentieon genomics tools [50]. Concurrently, somatic variations were identified using the Mutect2 module within Sentieon genomics tools. Variants were filtered using standard hard filtering parameters in accordance with the GATK Best Practices pipeline. InDels were categorized based on their chromosomal positions, which included intergenic regions, exons, introns, splicing sites, untranslated regions, and the 1-kb regions upstream and downstream. Additionally, their effects were classified, encompassing missense mutations, start codon gain or loss, stop codon gain or loss, and splicing mutations. InDel marker sequences were aligned against the reference genome using BLAST (v2.14.1) to ascertain their genomic locations, and the mapping was visualized using the TBtools software (v2.056).

### 4.5. Experimental Validation of InDel for Polymorphism

Initially, the resequencing data of the parents were globally aligned with the corresponding reference genomes to verify the coverage and reliability of the resequencing data. Subsequently, the resequencing data of the 62 offspring were aligned with the parental reference genomes to identify variations between the parents and hybrid offspring. InDels with lengths greater than 50 base pairs (bp) were selected. Integrative Genomics Viewer (IGV) was then utilized for visualization to identify parent-specific InDels. Finally, apple-specific InDels were chosen as markers. Primers were designed for the filtered markers using Primer 5.0, and PCR amplification was subsequently performed on both parents and all offspring. The PCR reaction system had a total volume of 20 μL. The PCR amplification conditions were as follows: an initial denaturation step at 95 °C for 5 min, followed by 36 cycles consisting of 30 s at 95 °C, 40 s at the respective annealing temperatures, and 45 s at 72 °C, concluding with a final extension step at 72 °C for 10 min. The PCR amplification products were then electrophoresed on a 2.0% agarose gel to assess the size of the amplified products. InDel markers that exhibited a single polymorphic band in both parents were considered suitable for hybrid testing. If the hybrid offspring produced two PCR amplification bands (two distinct alleles), each representing a single band from each parent, they were considered to be true hybrids.

### 4.6. InDel Marker Transferability

Using the same PCR screening conditions, we employed polymorphic InDel markers that were validated from the resequenced population (‘Golden Delicious’ × ‘Yan Zhuang’) to validate the hybrid offspring in the remaining three hybrid populations (‘Golden Delicious’ × ‘Jin Zhui’, ‘Fuji’ × ‘Yan Zhuang’, and ‘Fuji’ × ‘Jin Zhui’). Additionally, this process was used to evaluate the transferability of the InDel markers across different hybrid populations.

## 5. Conclusions

This study analyzed whole-genome resequencing data from the ‘Golden Delicious’ × ‘Yan Zhuang’ hybrid population and identified 51 InDel markers capable of accurately detecting hybrid offspring from distant apple–pear crosses. All 62 offspring were confirmed to carry genetic material from the paternal parent (pear), achieving a 100% hybridization rate. The highest identification efficiency was observed with markers InDel1-7, InDel2-3, InDel3-1, and InDel4-3, which, when combined, reached a 100% identification rate. These markers showed over 70% universality in other populations, including ‘Golden Delicious’ × ‘Jin Zhui’ (46 offspring), ‘Fuji’ × ‘Yan Zhuang’ (76 offspring), and ‘Fuji’ × ‘Jin Zhui’ (40 offspring), identifying 82.6%, 80.3%, and 85.0% of hybrid offspring, respectively. These InDel markers are efficient, easy to use, and accurate, making them suitable for rapid, large-scale hybrid identification, and essential for molecular-assisted breeding in distant hybridization of apples and pears.

## Figures and Tables

**Table 1:** Construction and cross-compatibility of distant hybridization populations between apple and pear.

Female (*Malus* × *domestica*)	Male (*Pyrus brestschneideri*)	No. of Flower	No. of Fruit	Fruit Set (%)	No. of Seed	No. of Seedling	Germination Rate (%)
Golden Delicous	Yan Zhuang	500	126	25.2	170	62	36.5
Golden Delicous	Jin Zhui	500	110	22.0	141	46	32.6
Fuji	Yan Zhuang	500	132	26.4	178	76	42.7
Fuji	Jin Zhui	500	103	20.6	134	40	29.9

**Table 2:** Summary of the re-sequencing results of parents and 62F1.

Ref. Genome	Sample	Clean-Reads	Map Ratio (%)	Q30	Depth	Cover Ratio	GC (%)
*Malus* × *domestica* ‘Golden Delicious’	‘Golden Delicious’	84,249,690	98.8	93.3	20.9	94.1%	38.2%
*Pyrus brestschneideri* ‘Dangshansuli’	‘Yan Zhuang’	75,193,854	95.9	93.0	19.7	91.2%	37.1%
*Malus* × *domestica* ‘Golden Delicious’	62 F1	46,661,113	98.9	97.3	19.4	89.4%	38.1%
*Pyrus brestschneideri* ‘Dangshansuli’	62 F1	50,255,070	94.3	97.1	18.2	82.6%	37.5%

**Table 3:** Identification of hybrid and InDel universality information.

Population	F1	True Hybrid	Hybrid Rate	Total InDel	Effective InDel	Universality Rate
‘Golden Delicious’ × ‘Jin Zhui’	46	38	82.6%	51	45	88.2%
‘Fuji’ × ‘Yan Zhuang’	76	61	80.3%	51	39	76.5%
‘Fuji’ × ‘Jin Zhui’	40	35	85.0%	51	36	70.6%

## Data Availability

Data will be made available on request. The sequence read archives of 64 samples (2 parents and 62 offspring) were deposited in the NCBI database (Accession No. PRJNA1221984).

## Supplementary Materials

The following supporting information can be downloaded at: https://www.mdpi.com/article/10.3390/plants14050646/s1, Figure S1: PCR validation and analysis of InDel markers; Figure S2: PCR validation and analysis of InDel markers; Figure S3: PCR validation and analysis of InDel markers; Table S1: Information of 340 InDel markers; Table S2: Information on InDel markers that are invalid in three hybrid populations.
