# Supplementary material for: InDel Markers for Identifying Interspecific Hybrid Offspring of Apple and Pear
Source: Plants (Basel). 2025 Feb 20;14(5):646. doi: 10.3390/plants14050646 (PMC11901437; doi:10.3390/plants14050646)
Supplement: Supplementary file 1 [file plants-14-00646-s001.zip › Supplementary Figures.pdf]

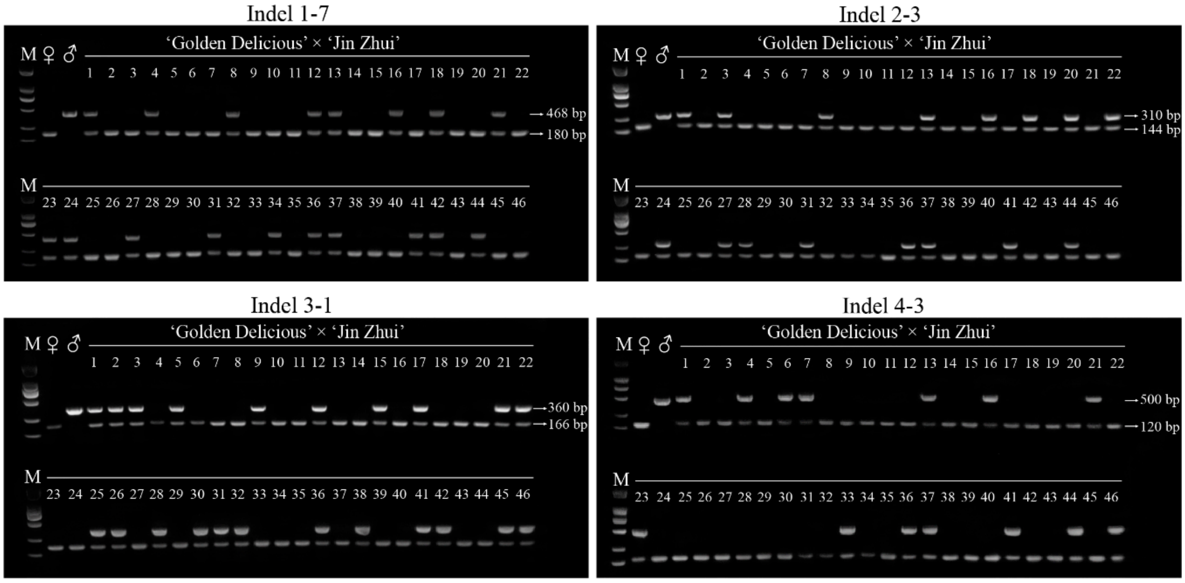

Figure S1. PCR validation and analysis of Indel markers. (A) In the 46 F_1 progeny of the cross between 'Golden Delicious' × 'Jin Zhui', PCR amplification of Indel markers Indel1-7, Indel2-3, Indel3-1, and Indel4-3 revealed that total of 38 individuals exhibited bands consistent with both parents, while the remaining offspring exhibited a single band matching the maternal parent. M, DNA Marker DL 2000. ♀, maternal parent, 'Golden Delicious'. ♂, paternal parent, 'Jin Zhui'.

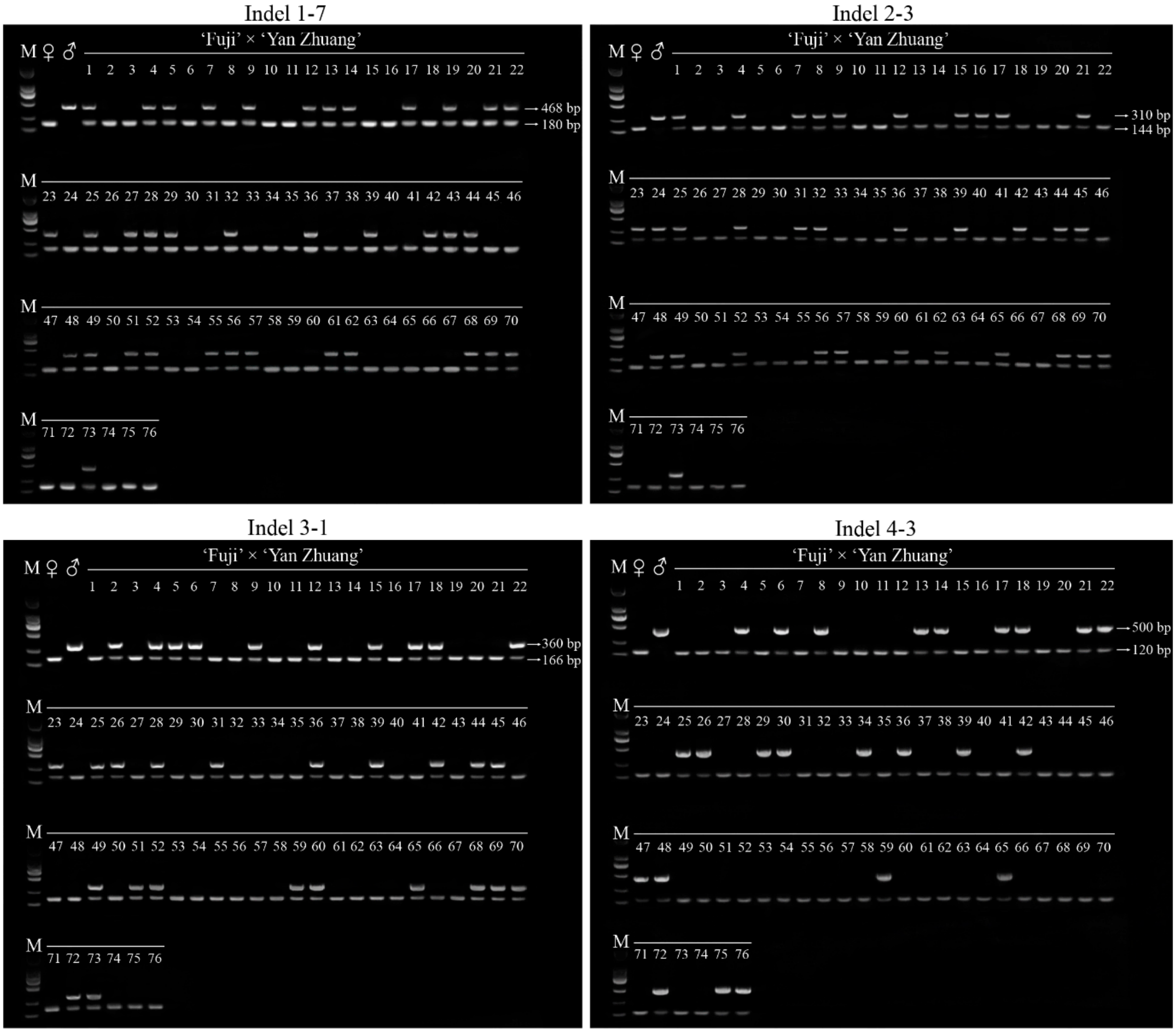

Figure S2. PCR validation and analysis of Indel markers. In the 76 F_1 progeny of the cross between ‘Fuji’ × ‘Yan Zhuang’, PCR amplification of Indel markers Indel1-7, Indel2-3, Indel3-1, and Indel4-3 revealed that total of 61 individuals exhibited bands consistent with both parents, while the remaining offspring exhibited a single band matching the maternal parent. M, DNA Marker DL 2000. ♀, maternal parent, ‘Fuji’. ♂, paternal parent, ‘Yan Zhuang’.

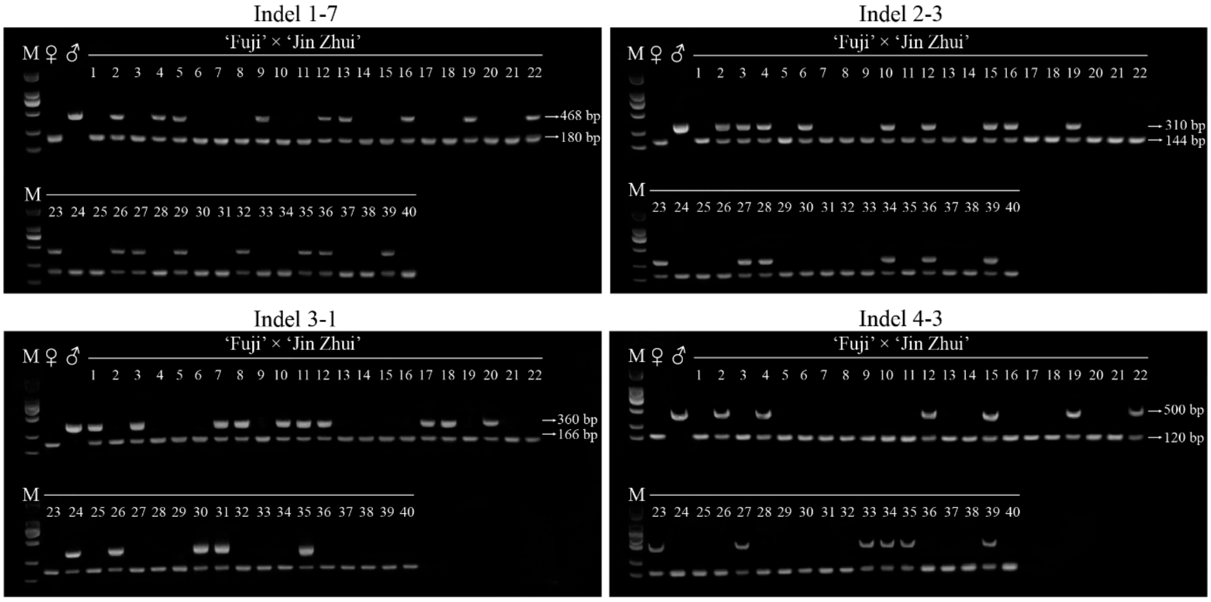

Figure S3. PCR validation and analysis of Indel markers. In the 40 F_1 progeny of the cross between ‘Fuji’ × ‘Jin Zhui’, PCR amplification of Indel markers Indel1-7, Indel2-3, Indel3-1, and Indel4-3 revealed that total of 35 individuals exhibited bands consistent with both parents, while the remaining offspring exhibited a single band matching the maternal parent. M, DNA Marker DL 2000. ♀, maternal parent, ‘Fuji’. ♂, paternal parent, ‘Jin Zhui’.
