# Supplementary material for: InDel Markers for Identifying Interspecific Hybrid Offspring of Apple and Pear
Source: Plants (Basel). 2025 Feb 20;14(5):646. doi: 10.3390/plants14050646 (PMC11901437; doi:10.3390/plants14050646)
Supplement: Supplementary file 1 [file plants-14-00646-s001.zip › Supplementary Tables.pdf]

Table S1 Information of 340 Indel markers (Bold font: 51 polymorphism markers).

Marker	Chr	Pos	Indel type	Size（bp）	Forward sequence	Reverse sequence	Product size （bp）	
							‘Golden Delicious’	‘Yan Zhuang’
Indel1-1	chr1	984234	insert	98	AGTTGGTAATAAGAACTCGAA	AGATTAGATTGGAGTTGAGAAA	98	98
Indel1-2	chr1	1295619	insert	107	GATGGTGCGGATTGCACCATT	CTTTTTTATATTATTTTTTTT	0	0
Indel1-3	chr1	1846552	insert	76	TGTCGATGTAGAGAAAGTGTTG	AACAAAACGTCGTATAAGGGAA	101	177
Indel1-4	chr1	2486763	insert	125	CCTTTACTATCGTAGTCCAAT	CAAATCAAAATTAAATGAAGAA	130	255
Indel1-5	chr1	3187656	insert	106	ATTTCTCAACAACTTTACGAT	CATTGAGGTTGTTTGATAGGA	106	106
Indel1-6	chr1	5633507	delete	100	AATTTTATTATATCAATCCCTA	ATAATGGAACACACTAAAGTCA	0	0
**Indel1-7**	**chr1**	**7835068**	**insert**	**288**	**CTAGGCTTTGAAAGTCATGGG**	**CTTGCTCATGTCGAGCTTAAA**	**180**	**468**
Indel1-8	chr1	9014275	delete	87	ACACTTATGATCGAACTGAGA	TGTGAAAGCTTTCACGAACATT	87	87
**Indel1-9**	**chr1**	**12299701**	**insert**	**171**	**AGGAGAGAGGGAGAGATTGG**	**ACATCATTCTCAAAGGGCCCA**	**132**	**303**
Indel1-10	chr1	15457021	insert	96	TCACTCGGGAACTTAGGGGAGC	AGTGAATGATTTAGTACAAATG	96	96
Indel1-11	chr1	21082000	insert	92	AGGGGTTTCGCTTTTGATCCA	AAAGGGCAACGAAGGAAGGCTT	0	0
Indel1-12	chr1	23085135	delete	62	ATATCAAAGGATGTTTGACTT	CTATGATCATCTGGTCGGATCC	257	195
**Indel1-13**	**chr1**	**24298100**	**insert**	**265**	**TAGACTCTTCTGTCGATCCTG**	**GTAGAATGTATGTGCCAAATA**	**178**	**443**
Indel1-14	chr1	26204838	insert	113	AATGATATGGTTCAGTACAAC	CTCTAAATCAATCGACACGTGT	113	113
Indel1-15	chr1	27213609	insert	124	AGTTGAAAGAATTGTTGATAAA	GTTCTCTCAATCTTTGCTTCAT	124	124
Indel1-16	chr1	28014977	insert	115	CAAATTCAAAATCTCTAATTGG	CTCTATGCAAAAAAAAAAAAAA	115	115
Indel1-17	chr1	29014982	insert	94	GGACATTTCTAATTTAGGTAAT	AACTGGAGAAATCTGTGAAGTG	180	274
Indel1-18	chr1	30271573	insert	118	CACTTAGAACCGGTGTGGCAAA	CTCGGAACTATTGAGCTTTCCG	0	0
Indel1-19	chr1	30604933	insert	99	CTATTGTAAAGACTAGAGATGG	GTTTTCTTTTTATTTTTCATTG	99	99
Indel1-20	chr1	31022535	insert	132	CGTGGCCTTCTGGAGATGTGTT	TATTACAAATAAAAGGAATGAA	155	287
Indel2-1	chr2	100949	insert	210	AGGACAGTGTGTGAGTGTCTTT	TATGGAAGAGGTTCAGGGGAGA	210	210
Indel2-2	chr2	1597704	insert	128	CAGACAAATTCCTAGGGGATCT	CCAAAATACAAGAAGCTCTATT	128	128
**Indel2-3**	**chr2**	**3207645**	**insert**	**166**	**GAGACAGTGACAATTTTCGCA**	**TGTGGGGTATGAGGGTAGCT**	**144**	**310**
Indel2-4	chr2	6690095	delete	62	TCATAAAAGCACCAGGGCAGA	GTGTCCGTACATGTTTGTAAGC	322	260
Indel2-5	chr2	8695978	insert	89	AAGGGTGGAGCCAGAATTTTTT	AGACAAAAGAATATGTAAGAAG	0	0
**Indel2-6**	**chr2**	**10700001**	**insert**	**192**	**ATTTGTGAGCAAGCAACAAAG**	**GATCTGGTTCCTCTACTTTCTC**	**120**	**312**
Indel2-7	chr2	11713112	insert	86	TTTTAAGCATCCACAAATCTCA	GAGAAGGAGGCCAGAACACTCC	86	86
Indel2-8	chr2	13717547	insert	113	TTTCCCTCAAACCAGGAGGCCA	TTCCAGCTCATCGACTTTAGC	113	113
Indel2-9	chr2	16780486	insert	82	GGAGGAGGGATAGGCCGAGCA	CACTCCAAATTTTCCATATCAG	0	0

Indel2-10	chr2	18790747	insert	108	CCTTGGTTCCTTTCTCTGGCT	TTTGGTAATGTTAGTAGGCAGG	108	108
Indel2-11	chr2	20801313	insert	170	GTTAGTTTGAACTGCTTGGAAA	TGTGGCTGAACGAAAAATTCAT	170	170
Indel2-12	chr2	23801620	delete	96	ACACCTTCGAAAGGTGTGTTCT	TTTCTTTGTTAAATGAACAAA	0	0
Indel2-13	chr2	25808272	insert	96	TGAAGTGTTGAAACCTAATTGA	TATACTTCCAACAAACAAGGGA	210	306
Indel2-14	chr2	26581726	insert	115	TGGCGTCTTTCTAAGCGTTTAA	TATCGCAGTGCTAAGAAAGATT	115	115
Indel2-15	chr2	27819611	delete	141	GGTGACTCTAAGTTGGTCATTT	CCAACGGCTACATAATAAAGAG	166	307
**Indel2-16**	**chr2**	**28824987**	**insert**	**419**	**CAATTAACAATTAGGGTTACAT**	**TCTTCTAACAATCTGGAGAAG**	**130**	**549**
Indel2-17	chr2	29828303	insert	106	AGGCTTTTTGATGTATTCATTT	CAAGTTCTCAAACTTTTCTTTG	106	106
Indel2-18	chr2	30438537	insert	102	ACTGGACTTCAATCCCCTCAA	TTCTTCTCCGATTCGCACATCT	0	0
Indel2-19	chr2	32443884	insert	83	CCTCTAGTTCTTGAGCAGATGA	AGATCAACATACTTTCTTCATC	0	0
Indel2-20	chr2	33451914	insert	87	GTCACTAGTGAGGATAAGTACG	TTGGTACTACCGCATCAAAAGA	87	87
**Indel3-1**	**chr3**	**1284966**	**insert**	**194**	**TGCACAGATTCGGTCTTAGCA**	**AGCGGCGTTCGATACTGG**	**166**	**360**
Indel3-2	chr3	3443552	insert	86	TTGTTCATAATCGGTTGGCATT	TTCATTTTGGGTTAATTCATGT	86	86
Indel3-3	chr3	6143818	delete	102	CTTAGTGAAAACTAAGGAAAGT	AGAGGCTCCTGACTGAACGATT	360	258
Indel3-4	chr3	7045250	delete	83	TGAGTTCCCAAAAACAAAACCG	GCTCATATTCGATCTCAACCAT	177	260
Indel3-5	chr3	8346286	insert	105	TCTGTTACTTTTCTTTGTTTCT	AAGCATGACCAAAACACTAGAA	0	0
Indel3-6	chr3	9247695	insert	90	GACATAAACACACCAAATAAAA	GATGAATATGAGTCAGACACGA	90	90
Indel3-7	chr3	9707251	insert	113	CACTTTCCATTATCATCAAACA	TTGAAGAAATTGAGAAGTGATA	158	271
Indel3-8	chr3	10719584	insert	117	AAGTTAGTCGTAGAAAGAGGTT	TACATACGGAATTGATGAAACA	0	0
Indel3-9	chr3	13873412	insert	110	CATTTCCAATTTAATAGGACTG	ATGTTACCTGTAGAAACTCTCG	110	110
Indel3-10	chr3	14326926	insert	100	CTTAAGCAATCTCCTTGAGCGT	ATGAATTGACTAACAATGCTCAC	180	280
Indel3-11	chr3	15678716	insert	97	TGCCTAGCATTTCACGTAGCAA	TTGGTTATTCAGATAGTGATTA	201	298
Indel3-12	chr3	16578950	insert	101	ATGATGACCTCGGCTTGCCGA	AGGGTCTTGATGTGCTGGGTGC	101	101
**Indel3-13**	**chr3**	**18708753**	**insert**	**184**	**CCCTTGTACATACATGTGTGT**	**GTGGACACTTCGAACCGAAA**	**140**	**324**
Indel3-14	chr3	19585110	insert	111	TTCTTGAACTGGGCTGGAGGGT	AAGCAAGAGTATCCCATATCAT	0	0
**Indel3-15**	**chr3**	**21159480**	**insert**	**228**	**TTTTAGGTGTACTTCGTTCTTG**	**CTTTACGTGAACAGCACAATA**	**180**	**408**
Indel3-16	chr3	23159930	insert	93	ACTTCTCTCTATCTCTGCCTTC	AAGATCAAGCACAATCTTAATG	93	93
Indel3-17	chr3	25574638	delete	100	AGCAACGTATTTACTTATGTGA	GACTTCAACAATTCTTCGCTTC	0	0
Indel3-18	chr3	27376096	insert	99	AAGACTCTTCCTTTATGAGTAT	GCATCACTATAACAAAGTACCG	203	302
Indel3-19	chr3	30081248	insert	86	AGTTGTTCTTCTCGCTAACATA	AACAACCCCTTCTCGTATAGCT	99	99
Indel3-20	chr3	32193404	delete	103	GAAGCCTTCCTATTTTACTGGT	CATCTTTACTTTCACTCCATGT	441	338
Indel4-1	chr4	1213738	insert	116	GCCACATGTTTTCTTTCTGTCT	TCTTCACGTGACAAAGAAGGCT	0	0
Indel4-2	chr4	3298512	delete	127	GGAGTCTAAATTGTGGGTGCAA	GTTTCAAGGGGATCGAAGGCTC	350	223

**Indel4-3**	**chr4**	**6605032**	**insert**	**380**	**ACTGCAGAGCGCCCTTTT**	**GTCCAGCAGGCACCAGAA**	**120**	**500**
Indel4-4	chr4	7211932	insert	102	TTAAGAGTGTTACTAGACCCAC	GGCTTGAAACTTCAACCTAATC	0	0
Indel4-5	chr4	8161431	insert	132	CAATAATGCATGCACATGAGTA	AAGGGAAGCCCCCTATAATCAT	132	132
Indel4-6	chr4	9022004	insert	87	AAATCCATAAGCATCTCTTAAG	TGCTACCAAATGTGAAAGCATC	152	239
Indel4-7	chr4	11823084	insert	165	AATGTTTGCCGAACAGATTGGG	TGCCTCAGCACATTCATCAGTC	175	340
Indel4-8	chr4	12133938	insert	88	CGTAAATCCTGAAGGATCAAGA	ATTTGTGGATGGGTTGTTGATC	88	88
Indel4-9	chr4	13240204	insert	97	GGCGTTTCATCATTTAGTTCCA	TCTTTTTCTACCCGTGCCTTAT	0	0
**Indel4-10**	**chr4**	**15665776**	**insert**	**214**	**AGTAGTACCGAGAAGAAGTCA**	**TATTCTAGCATCAAATTCATG**	**136**	**350**
Indel4-11	chr4	16776708	insert	105	TTCTTCCAAGAGTATTCGATGC	GATCAAATACTGGAAAGGTTAG	105	105
Indel4-12	chr4	17061685	insert	89	AAGGAATGCATCTCCTTAAAGA	GCTCCAGTAGCCTAATTTAGAG	140	229
Indel4-13	chr4	17861687	insert	119	AGACATGTTAGGGTGTTTTTAC	TATCTCTAAAACTCAACAACGT	119	119
**Indel4-14**	**chr4**	**18965760**	**insert**	**165**	**CTCTCACCTTATGGGAGAAAA**	**AGCTCAAGGGGAGTGTAATG**	**140**	**305**
Indel4-15	chr4	19265766	insert	87	GGGAAGAAAAATGGGTGCATAT	CGTTGAAACAGCAAGTTTGAAA	87	87
Indel4-16	chr4	21168393	insert	89	AAATTTAGTCACTTTGCACTTT	TGACGTCTCACCTTTGTGGTAG	0	0
Indel4-17	chr4	25223246	insert	84	TTCTATACAATGGGAAAACGTT	CTCAAATCAAGGGTAACTAAGT	0	0
Indel4-18	chr4	26184502	insert	97	TGGACCAAAGCTACATTTCCAG	GGAGAATTTTTTTCCATTGTCA	97	97
Indel4-19	chr4	28091330	insert	95	CATTGTGTTACATTAAACCCAA	TTCATGCGCATAGCTTGTTTAA	120	215
Indel4-20	chr4	29932000	insert	112	GTCTTGTTGATAGAAAAGTCAC	ATCCTTTTGGGTTTTGTAGTTC	156	268
Indel5-1	chr5	1493668	insert	88	ATAATGATCCTTCCGATAAATC	TCCGCTAGATTAACGCTAGAA	0	0
Indel5-2	chr5	2306529	insert	91	ATGGTTTGGAAGGTTCACTGAA	CTTCACTAAGAGCATGCATGAA	91	91
Indel5-3	chr5	4710906	delete	141	TAGACCGGATACAATTTGTCAG	GGGGTCGTGACCCTGTTACATC	410	269
Indel5-4	chr5	7217312	insert	207	GGATACAATAGCCATGAATCCC	AGACAGGCAACTATGGAGGTTT	207	207
**Indel5-5**	**chr5**	**9019863**	**insert**	**174**	**TGAATGGCAGCAGCACGA**	**GCTCCTCGCCAGTGAGAA**	**177**	**351**
Indel5-6	chr5	9921383	delete	110	CCAGAGGGATTGATTTGAAGAA	ATCTTCCACTGAAAAGTAGCAC	0	0
Indel5-7	chr5	11921934	insert	95	CCTTAGGTGATTCTTTTGATAT	CTTCAATTTGTTGAAAGCATCA	110	205
Indel5-8	chr5	12625098	insert	92	GCTGTCCTTGGATTCTTCCTTG	TTAAGGACGGTGACCAATGATC	132	224
Indel5-9	chr5	13225110	delete	85	GAAGTTGGAGAAGATGGCTTAC	AACAGCTGCTGCTCGAAAAATA	85	85
Indel5-10	chr5	15230016	insert	114	TAAGTTTTTACCCGCATAACCG	GGTTATTAGCAGACTGATGCGT	0	0
Indel5-11	chr5	17630598	insert	84	TGCCATAGGCTCTTTGATCATT	TCATTCACAGCAAGCTCCACGA	158	242
**Indel5-12**	**chr5**	**19255475**	**insert**	**384**	**GGCCTCTTGTGTGTTTTTTTG**	**GGCATTTGTAGAGCCAAAAAT**	**130**	**514**
Indel5-13	chr5	21365037	insert	94	TCAAAATTCCAAGTCTTACCC	GGGAACTATAACGAAAAGCTCCC	0	0
Indel5-14	chr5	23171180	insert	106	AAAAGCCTTTTGCCTTCAAAAC	TCAAAATGGGTACATTTAGCAAA	178	284
Indel5-15	chr5	25677330	insert	82	CTTCTACTTCTGCAAAATCTGT	GGTGATTGATTTTGAAGAATGG	166	248

Indel5-16	chr5	27101065	insert	97	AAGTTGGTGTGTAGAGCCAGA	GCCGCCCGCATTTATTTACAC	190	287
Indel5-17	chr5	28921129	insert	116	GCACCTTGCTAGTAACTGCAAA	ATGATGACTCGTCCTGACATT	116	116
Indel5-18	chr5	31025854	insert	109	TTATTAATGACGTCTGTTCTGAT	TGTTGGGGATTGGATACCGAAT	0	0
**Indel5-19**	**chr5**	**38225859**	**insert**	**229**	**ATTTGCTGCCCGCGGTATAAC**	**CCACAACCTGCGAGCTCACTAC**	**150**	**379**
Indel5-20	chr5	41229195	insert	118	TGACGTATGATAGAACTTTGTT	ACTATTACGTGACGAATGAACC	118	118
Indel6-1	chr6	2532061	insert	91	GGAGGATGGAAGAAATAAATGA	CTTTCATACCTGCGACTAAGGC	91	91
Indel6-2	chr6	4736427	insert	108	TTTTAATTGCATGTCCCGGTCT	GACCATCATCGTGTGATTCATC	133	241
Indel6-3	chr6	7058843	insert	359	AATATGTCGAGTGCTTGTTTGT	AGTTTCTGAAAGGAGAAATCGA	0	0
**Indel6-4**	**chr6**	**9158845**	**insert**	**467**	**TGAGCTATACCTTTCGTGCAA**	**TCCCACCTTACGTACGTACA**	**110**	**577**
Indel6-5	chr6	11358848	insert	94	CATCACTCCTCTCTCTCTTCCC	TTGATGAGATGAACACATCCAT	0	0
Indel6-6	chr6	13061418	insert	92	TAATCCATCCAATCCGTCCACC	TGCATTTAGGTGGCAGCTTCC	167	259
Indel6-7	chr6	13773265	insert	90	AATGACCTTCGAGGTTCTAGAC	ATTGATCACATACGCGCAAACT	204	294
Indel6-8	chr6	15674917	insert	189	GAACCGTCGATGCTTGATCTT	AAGTATCTTATGTCATCAGGGT	189	189
Indel6-9	chr6	16219518	delete	66	AACCATGATATGCTCAAGGTGT	ACGCAACGCTTTCTGAATTAGA	66	66
**Indel6-10**	**chr6**	**18280724**	**insert**	**214**	**GGATCTTCCATGGACAACCCA**	**GGAAAAGGAAAGTACCAGAC**	**148**	**362**
Indel6-11	chr6	19187133	insert	99	TTGCAAGAATGACAATGTTATT	GGAGGTTCTAAGTAGAATATGA	99	99
Indel6-12	chr6	20087548	insert	101	GTTGGTTCCTTGTAGAAGTTTAC	AAACTAAATGAGGAGTTCATAA	0	0
**Indel6-13**	**chr6**	**21187662**	**insert**	**311**	**AAGAGTGTGTGAAGCTTTTGA**	**TTCCACCTACTAGAAGACGAA**	**160**	**471**
Indel6-14	chr6	23388026	insert	135	ATAAGGAAGACTTTGGATGCGG	GAATTTACCCATGTTTTGGTAC	135	135
Indel6-15	chr6	27988700	delete	88	ATTCGGAGAGCGGTGTCTCTTC	TAAAGGAACAGTACCACTGCTG	256	168
Indel6-16	chr6	28709926	insert	111	ATTTTCCGGTGGGAGTCCAATT	CATCGTAGATTTCGCCGTCGTT	111	111
Indel6-17	chr6	30092044	delete	90	ATAACTCGGCCTCCTTACAGCC	ATATTTCTAGAAACTTCAGGTT	0	0
Indel6-18	chr6	31023025	insert	105	ATCCTTCAAAAGGTGAGTTATG	GGATATTAGACGATATGAGGTG	0	0
Indel6-19	chr6	32224697	insert	129	TAATTACCGAAATGTCCTTCTC	CGAACTCCTTGAACACCAAAGC	129	129
Indel6-20	chr6	34248062	delete	147	AAGATTCAAGCACGCCATGATT	AATTTATGCCTTTTGCACCGAA	310	163
Indel7-1	chr7	1433261	insert	114	AGTGATCTAAACTGTTAGCAGT	GAGGCTCTTAGCCTTGACCTAA	114	114
Indel7-2	chr7	1900784	insert	107	GCCGGTGGACGAAGAGAACTCT	CCGGAGAAGTAGGACATGAATT	0	0
Indel7-3	chr7	2850786	insert	191	TTGGCTTTAGCAGCTGCTGTA	AATCTTCTCTCTCTTCGTCTTC	129	320
**Indel7-4**	**chr7**	**3206685**	**insert**	**200**	**TGGATAAGACCGTCAGAGGG**	**AGGCGGTCAAGCATCAACA**	**137**	**337**
Indel7-5	chr7	4016686	insert	154	GACCATTGCACACCACACACAC	GGGTTTAGGGTGAGTGTGGAG	0	0
**Indel7-6**	**chr7**	**8076464**	**insert**	**188**	**GCTGCCAGCAGTTCTTGT**	**CACCAACAAGAACTGCTGG**	**170**	**358**
Indel7-7	chr7	10213741	insert	159	AAGAAGTGGGATTTGTAACATT	GCCTAAAATTACCAAGTCATAT	177	336
Indel7-8	chr7	15418988	insert	103	CCCAAATTTGCTAGCATTCACC	CAATTCACAATCGATCGTTAAG	0	0

Indel7-9	chr7	18526093	insert	115	CCACCACTCATTCACCTTATAA	TCGAATTCAGAACAAAGCATTA	115	115
Indel7-10	chr7	19254073	delete	92	GTCAACCTTCAAGAGTCAAGCC	TGGGCTGAAGATTTCTTCAAGG	92	92
Indel7-11	chr7	20060442	insert	94	TGATATGGGATACTCTTGCTTT	TCCCAACATGATTACTTTCTCG	180	274
Indel7-12	chr7	22060447	insert	93	TTGTTTATTTGATTGGTTGAGT	ACCCAAGGAAAAATGATGATTC	93	93
Indel7-13	chr7	23175127	insert	83	ACGGAATAATTGTCCTACTGCT	CCTAGCATGAGGCCTTTTGGGA	0	0
Indel7-14	chr7	25485265	insert	92	AATGCAGACTGCACAATCAAAT	CAAATCCTCATCATTGTGTTGG	0	0
Indel7-15	chr7	26285504	insert	80	GGAGGGGAAGTCGAAGAAGAAG	CATTACTATTTAGCTTATGAGTA	123	203
Indel7-16	chr7	27186277	insert	92	ATCTAATTTGGTTAGGGCTGGA	CCTTGAATTCAAGGTGGATACT	92	92
Indel7-17	chr7	28495847	delete	87	CAAACCAGTTTATTTCAACCAT	TTTAAACCCCCCAATGGAGACA	362	275
**Indel7-18**	**chr7**	**29197507**	**insert**	**156**	**TGTTATCTCTACACTTCGACC**	**TGAACACAAAGTACAGTCTG**	**200**	**356**
Indel7-19	chr7	30898354	insert	87	CCTACGTCCATATTTTTCTCTC	CTCGGGAGAATGTTTACTGGAC	0	0
Indel7-20	chr7	32901360	insert	121	AAATGTGATGCTAAAAGGAAGA	TGAGCGAACAAACAACTTTTCA	121	121
Indel8-1	chr8	1002520	delete	153	TAGGAAAACGCAGTTGGATTAT	GCGAATGCAAAGATACAAACTC	390	237
Indel8-2	chr8	2794277	insert	105	ATTCAAGTGTGCTTTGGAACTT	ATTCATGTGTTTTCTACTTCCC	105	105
Indel8-3	chr8	3125046	insert	110	AGCACTATTTTTTATACAAGCA	TGAAGTTCTTTCCAAGGCTTATA	110	110
Indel8-4	chr8	5685588	insert	111	GATCCAATGTGCCTCCAAGTG	AACGGCATCTCCAACTTCTTGA	0	0
Indel8-5	chr8	7858492	insert	230	CACCAGAGTTTTTTGTTTCTTC	TACTTTATGGCTTCGTCACATT	125	355
Indel8-6	chr8	11458503	insert	88	TCAACTGAGACGGTTTATAAAT	TCAAGGAAAATAGCATCATAAG	0	0
Indel8-7	chr8	12158505	insert	123	TGCATGGGTGGTTAACATTTGT	CAACATCCAAACCAGACTTTTC	123	123
Indel8-8	chr8	13261397	insert	113	TTTGGATAATGCCGCACCATTG	TTCATCTGATACTGTGGACAAG	178	291
Indel8-9	chr8	14062420	insert	90	AGCATATACTCAGATGGGGTCG	CTCATGCGTTCTAAATGGCTTG	90	90
**Indel8-10**	**chr8**	**14367410**	**insert**	**219**	**CGTCCGTCCTTTGCACCT**	**AGCCTACTCCCTCACCCC**	**143**	**362**
Indel8-11	chr8	15438181	delete	91	GAGAGGAAGAGTCATGATGGGA	CAGTTTCGTTGCGCAATCTTCA	0	0
Indel8-12	chr8	16016856	insert	112	GGATCAGTTAGAGCCTCATGCA	CAAGTGGTGATTATTCACATGG	112	112
Indel8-13	chr8	16787587	delete	107	TCCTCCTTCGGGGCATTAGGAA	TTTGGGGCAGTTTTGAGTCTTT	301	194
Indel8-14	chr8	19579975	insert	147	GGATGTTGTTCTAGAAGCTTA	CCCGTAGGATTTAGCATATTTC	144	291
Indel8-15	chr8	20080008	insert	140	GGGTAATGCTAGAGAGACTTAA	ACTCAAAATTCTTCAAAGACAT	0	0
**Indel8-16**	**chr8**	**21283372**	**insert**	**114**	**GCTTCTCCTTATCCTTGTTGCT**	**GGCTTCTTCTCTTGATGTATTT**	**166**	**280**
Indel8-17	chr8	23288741	insert	175	TGTAAATATGGCGTAAACGCAA	GCTGCCTTTTTTTACTGTTAAT	175	175
Indel8-18	chr8	25689927	delete	95	CCATAAACAACAACTACAGAGT	GGAGTATCTCTGCGTCAAGGTA	288	193
**Indel8-19**	**chr8**	**26790463**	**insert**	**153**	**GTATTTTAGAGGGTAAAGTTGT**	**TTGTTTGGATTAGCCGACTCA**	**188**	**341**
Indel8-20	chr8	28243878	insert	203	CCGACTAATATTCTGGATACAT	TCCTTTGGATCACATGCATATT	203	203
Indel9-1	chr9	1393469	insert	117	TTGCATACTGGATGATGAGAAG	TCCTGAAACTTATGTGGCTTGC	0	0

Indel9-2	chr9	2393673	insert	130	AATTCATCCGATCTAACGGTCA	GAGGTGTGCGACTTCACATTAT	148	278
Indel9-3	chr9	5093738	insert	86	CAGGAAAGGAGCTAGGGATAGC	CCGACAAAAGGAAAATGCTCAA	0	0
Indel9-4	chr9	7993739	insert	99	GGATTTTTTGGAAGACAAATTG	GCAACTCAAAAAAAAGAAAATG	163	262
Indel9-5	chr9	9397646	insert	129	ATTTGGAAGCTCAAGTGGTTAA	GATAACTAGTATCACGTCACCG	129	129
Indel9-6	chr9	11097740	delete	84	ATATTTACTTCAACGCAGCCTG	AACTCTTAAAAGCCCCTGTTTT	0	0
**Indel9-7**	**chr9**	**12198591**	**insert**	**212**	**CAGCTTTCTGCACACATCATT**	**AGGAGAGAGGGAGAGATTGG**	**169**	**381**
Indel9-8	chr9	13200706	insert	103	TTGGCAAAGGCAATTATAGACT	AGAAAGCATTCCTTATCAAAGA	142	245
Indel9-9	chr9	15016712	insert	113	CCCCCCCTTAAATTTTCTAGGT	ATACGGACCTTTCCGTATCCTT	113	113
**Indel9-10**	**chr9**	**16701674**	**insert**	**304**	**CCGAATCTGGGGAATTTTTTT**	**AAAAAAAAGCCGAATCTGGGC**	**140**	**444**
Indel9-11	chr9	19022020	insert	105	GCATTTCATAAGTTGTGGATTC	CCCTCAGATTTTCCTTAAGTTA	105	105
Indel9-12	chr9	21005242	insert	98	AATTGTTATTGGGGAAATTGCC	CTCGTTATCTAGGGAGGATTGA	0	0
**Indel9-13**	**chr9**	**22328617**	**insert**	**327**	**GTGTTTATTAGCATCCCTAGT**	**TTGAATCACGACGTACTATGT**	**115**	**442**
Indel9-14	chr9	23229129	insert	104	CCGATGATAAGGGCTTCATATT	ACGTTATCCGGTACATGCTTAC	104	104
Indel9-15	chr9	24337144	delete	108	AAAACTATCCCGAGGCCTTTAA	CCAAATCACATTCAAGGTAATC	444	336
Indel9-16	chr9	28019014	insert	121	TGAGAGACTTTGCAGGTTTGTT	CCGACTTTCCAGATTTGGATGA	160	281
Indel9-17	chr9	29140541	insert	88	AAATATCCAAGACAGAGTGGCA	CTCAAAGAAAGAAGAGAAATGC	0	0
Indel9-18	chr9	30040542	insert	103	AGTGACCCATGTGTTTTCGGTA	GATTTTCTCTAATGCCCTGATT	103	103
Indel9-19	chr9	31341818	insert	152	CCGATATATTTGCAAACGCTAT	GGTTGTTCAAAGCAACTCTTAT	152	152
Indel9-20	chr9	32468891	insert	117	TCGAGTTCTTAGTGACATCATT	ACCAAGCAAGAGGGAAAAAATT	117	117
Indel10-1	chr10	820488	insert	97	TACATCAAGTATGGATGAAAAC	TAGCAATATGCAATGTGAGTTC	0	0
Indel10-2	chr10	1049118	insert	108	AATGTGAGTAAAGGTTGGGCAT	CTGGCGAGGTAGAAAGCACATG	201	309
Indel10-3	chr10	2286113	insert	93	GTGATTAGGTAGGAATGTGGAA	ATTCTTGGAGGTGGTGCAAACA	168	261
**Indel10-4**	**chr10**	**5518055**	**insert**	**141**	**GATAACAACAAAGCTGGACTA**	**GTCCATTTGTGTCCACCTCCA**	**170**	**311**
Indel10-5	chr10	6359084	insert	110	TGAACTTGTAAAACTTGACCCG	TTAAAATGCGTAAGTTGCGCAT	110	110
Indel10-6	chr10	8565104	insert	77	AAAAGGAAATTGAAATGCCACT	TACCTGTCGTACCCTAAAACAT	0	0
**Indel10-7**	**chr10**	**10177043**	**insert**	**280**	**AATTTGGGGGTAGATTGCCAT**	**AGGTAGAGATGGGAATATACA**	**230**	**510**
Indel10-8	chr10	12405367	insert	86	TGTGAATTCATCCACTTCAAGT	GATTGTTTCTAAAGCAGCGAGG	198	284
Indel10-9	chr10	17531957	insert	131	TTTAACCTATCTCGTCCCTCCA	GTGAAATGTTTTGAAGATGTTG	0	0
Indel10-10	chr10	18709588	insert	128	GGAGTTAAAATGAGTGAGAGAT	TACATTGCTCAATTGAGGTATT	128	128
Indel10-11	chr10	19639208	delete	94	CAATTAGCTAGCTTAATTGGGG	GGTAACAATTCAAAGTCCATCT	289	195
Indel10-12	chr10	19803774	insert	76	TGAAAAAATGCCGGAAAAGGAC	AAACCAATGTTGTCCAAACAAG	76	76
Indel10-13	chr10	22076747	insert	137	CCGAACCCAAAGGAAGAAGAAG	GAAACTCAAATCTAGTTTCCGA	137	137
Indel10-14	chr10	24602180	insert	98	GACTTGCAAGATCCAAGCCATT	TTCAAGAATAAGCCTGTGGAAA	0	0

Indel10-15	chr10	27327550	insert	85	CTAGTGCATGAAATAAATGTGG	CCTCTATAAAACCAATTGGCAA	85	85
Indel10-16	chr10	29886348	insert	81	CGACTTGTTAAGGACCTGTTAA	GGAAATGGTAGGGCGGACAAAA	139	220
**Indel10-17**	**chr10**	**30369527**	**insert**	**381**	**ACTCACAAGTCCTTCCGCA**	**TCCACAATGTCCCCCAGA**	**120**	**501**
Indel10-18	chr10	32405008	insert	64	ATGTAAAGGACCAAAATAACCT	GGAACAAGCATCAAATGAAAAG	64	64
Indel10-19	chr10	34125297	insert	83	GATAAGTCTGAGATGATTACGG	ACGAAAATTGCGACACAAATGAC	0	0
Indel10-20	chr10	37578195	insert	121	CAAGGCTCATCCTTAAGTCCTT	CACTTCATCCATCCAGCTTGTA	133	254
Indel11-1	chr11	509559	insert	84	GTCCCATAGTCGCATCTTCTAA	GCTGATTCATCTCAGGTATTGG	0	0
Indel11-2	chr11	734001	insert	110	TAGAAGCGTTGTCGCCCAGAAG	CGGGCCCTTATATTTTAGTATT	0	0
Indel11-3	chr11	858770	delete	161	GTAGATACCTTAAATCACCGAT	TTTAGGTGCTGATTACTTTCGG	161	161
Indel11-4	chr11	1018213	insert	92	AGTCTTTGTATATTAGGTTAAT	CTTACCTCTTCAACCATAGCCA	180	272
Indel11-5	chr11	2358376	insert	112	TGCCGAGCATTAGCCACTCTAT	CTTGGTAAAACTTCTGAATGCC	190	302
Indel11-6	chr11	3245675	insert	52	GCTTCCAAACCAAAAATAAAGT	GAAACCCCCAATTGATTTGATA	52	52
**Indel11-7**	**chr11**	**4467321**	**insert**	**231**	**CCAAAAGCATGTGCTGAGGTA**	**TAAATTAGGCAAGTTAGGCAA**	**142**	**373**
Indel11-8	chr11	5749025	insert	101	ACATGGTATCAGAGCCACCAGG	ACCAACAAAACGGATCTGGAAT	0	0
Indel11-9	chr11	6789032	insert	54	TCTCAGAAGACGAAGAAGCTGA	TATATGCCATCACGCTGCAGCC	54	54
Indel11-10	chr11	7765780	insert	107	TTCTGTGATTTTGATCCGTGCT	GACATTCCATCAAACACATAGA	156	263
Indel11-11	chr11	8936458	delete	84	CGATAGCCGGAGTGAAAAGTTA	CATCAGTCAACACAATCTATGC	84	84
**Indel11-12**	**chr11**	**10702515**	**insert**	**205**	**TTCCTCTCCTTATACATCTCTC**	**AGAGATCACGAGATGACGAA**	**162**	**367**
Indel11-13	chr11	11389566	insert	100	GGTTCGGGTAGTTCGAATAATG	GAAGCGTCGATTAATTCCCGAC	100	100
**Indel11-14**	**chr11**	**12055834**	**insert**	**185**	**AATTGATTTTCAGGTTGCCCC**	**GCTATATCCATATCCCTAACT**	**190**	**375**
Indel11-15	chr11	28182443	insert	93	GCTCCTAGCATTCAAAAAGAAA	CCAACATGATTAACCACACTAT	0	0
Indel11-16	chr11	29279014	insert	111	AATAATGCCATGTAGAAAGGCA	GGGTGAACAAGTGGATCTTTCG	205	316
Indel11-17	chr11	30773204	insert	106	AAGGTGGTGGAAGTACAACAGA	CACAACAAATCTCCCTTTGAAC	191	297
Indel11-18	chr11	32345631	insert	52	TACAGTTGGAGCCTCGGAAGTG	AACAATTCAGACTGCGAGTGGT	52	52
Indel11-19	chr11	35324317	insert	112	AATTTGACAACCCTTAGAGACT	GGTAGTCACTCCAAGTCTTATG	0	0
Indel11-20	chr11	39002105	insert	106	TCCACAAAACCCATAAGACCAT	TTTACATGGTATCTTTCGCCTG	0	0
Indel12-1	chr12	639786	insert	91	TTTTTTTGAAAGCAACGCAGTT	CACAAACGCCTCAATATATCTG	131	222
Indel12-2	chr12	805392	insert	96	GTTTGGTAAATCCAAAAGAACA	GGAGAGCTTGAACCTATGTACT	178	274
Indel12-3	chr12	1011223	insert	158	CCATGGACAGTGTAGGAAGAAG	CTAGTGATCCAACGATCCTTCT	158	158
Indel12-4	chr12	1811254	delete	136	ATGCACAAAATCAGTGATGACT	ACACGTGTTGACATCAGAAGCC	136	136
Indel12-5	chr12	2331555	insert	96	AGTATCGCGTCATGCAGCTTG	GTGGGACCCAGTGCTAAACTAC	0	0
**Indel12-6**	**chr12**	**3418896**	**insert**	**154**	**CATGTGGTAGACAAGCCAAA**	**GACTCATTCCTCTTGTAATCG**	**178**	**332**
Indel12-7	chr12	6415647	insert	67	TCAATATTCTCGCCTCTAGGGC	TATTAGTTGGATGTACTCGAAC	67	67

Indel12-8	chr12	9583607	insert	118	CATGAACCTGATGTCTTGCTCC	GCGACATGGTCAGGGTTCATGA	0	0
Indel12-9	chr12	12911745	insert	161	TTAATTACAAGTTGCCACTCAC	TACATAGCAAGCTATTCACTTT	161	161
**Indel12-10**	**chr12**	**15990290**	**insert**	**122**	**CCTTGTTAAGGTGGCTATTT**	**TTATGAGAACATTCGCCGGAG**	**165**	**287**
Indel12-11	chr12	17899820	insert	95	CTAATTTCTCTCCAGATATCCT	TCCACGAGATTTGAATTTAAGA	0	0
Indel12-12	chr12	20026673	insert	84	ATGTTAGCATCTTGTCGGTGCA	CCATGGTTACACAGTTAGCAGT	160	244
Indel12-13	chr12	36140073	insert	141	CAGAAAAGATATGAAAACGTCG	GATGTACCCTCACGAGTTTTT	141	141
Indel12-14	chr12	20924421	delete	182	ACCCTCGAAAGAATTTTTTTCA	CGAAATAATTGCAAACACTCTT	182	182
Indel12-15	chr12	21984686	insert	88	TGGAGTCAGATTGATGCCAGTC	CTCCAGAGCTCTTAATCTTCGA	0	0
Indel12-16	chr12	23944172	insert	103	TTAACATGGGTCTCAATCACTT	GGCCTCTATCTCTTTCTGAAGT	103	103
**Indel12-17**	**chr12**	**26183652**	**insert**	**112**	**GAGAACCATAGAAACTTACGG**	**ATCAGCGCCTACAACAATTAT**	**188**	**300**
Indel12-18	chr12	28420143	insert	223	ATAAACCTACCATAACCTTGCA	GAAGATGAATTGGACTTTGTTG	138	361
Indel12-19	chr12	30720970	insert	89	ACATCACTCCAGGACCTTCAAT	AGGCTCTATGTAACTGTGCTTA	0	0
Indel12-20	chr12	32857809	insert	210	AACAACTCTGTTCCTTAAGTGA	AAAAGCATATTGTAGGGCCCTC	210	210
Indel13-1	chr13	903448	insert	91	GAGTGCTGCTATCCTCAATCTT	CTCTTGCATTTGACGAATTTTC	0	0
Indel13-2	chr13	1185544	insert	157	CCTCCTCTTTAGAACCTCCTCC	AGATGATTGCAGAGAAAGCTCG	157	157
Indel13-3	chr13	1437879	insert	144	GCTACTGACTTCTGCGTCTTGC	ACATCTTCCGTTGGCATCAACA	122	266
Indel13-4	chr13	1990264	insert	92	TGGTTTGTTACTGCTATGCCTC	TCGGCTGTGAAGATTGTTCCAT	210	302
Indel13-5	chr13	2789251	insert	79	GTTTGCCGAAAAGCACGAACTT	AAAATCAATAAACGCGTCATGT	79	79
Indel13-6	chr13	3876473	insert	84	TCAAAGAACAAACTAGTGAGCA	CGAGGTGGGTTTCTCTTTGTTT	0	0
**Indel13-7**	**chr13**	**6192036**	**insert**	**190**	**TTCAAGCTTTGAAGGGAACAG**	**CTCTCACTCACACAACCATAA**	**115**	**305**
Indel13-8	chr13	10192277	delete	124	CCCGAGGCCTTAGAATGAGAGG	TCCCTTAGTGGTGGCTCATACT	0	0
Indel13-9	chr13	12163109	delete	107	AAATGCTAAGAAGACTCTCTCA	TAAACAGTATCTGTGACGACGC	107	107
Indel13-10	chr13	18823950	insert	182	GTGCTTATCAATTCTTCATTGT	TTTAGGTAATCAGCAGCCAAAT	182	182
**Indel13-11**	**chr13**	**20207387**	**insert**	**147**	**ACCTCCCATATGCCAGTGC**	**AGGCAGCAGGAACAAGCA**	**167**	**314**
Indel13-12	chr13	23332956	insert	179	GTGTCATGTGTTCAAGGCTAAG	AGCCCGTAAGGTTTTAGAAACA	179	179
Indel13-13	chr13	28817373	insert	98	GAATGAAATGGGCATGAGTTTC	ATTTGATTTACTCTTTGGTGTT	0	0
Indel13-14	chr13	37111199	insert	289	TTAGCTGAGGATGAATTAGCAC	TTTCGTTGTTTTCACTGCGGAC	289	289
**Indel13-15**	**chr13**	**38861242**	**insert**	**209**	**GCCTCAGGTCAAAAGGACTA**	**TTTAAGCAATAAGGGAATCCA**	**130**	**339**
Indel13-16	chr13	33811563	insert	68	CAAGTGCGGACAAATCTGGAAT	TTCATCATCGAATCCCCAATGG	68	68
Indel13-17	chr13	36685527	insert	99	CCAATCCATTTAGACCGTAACT	ACAAAACACCTCTAGGGATGTC	140	239
Indel13-18	chr13	38006047	insert	82	TTTGGTCCTTGGCACCTATTG	GTTGTCCTTAGAGGTAAGTGA	0	0
Indel13-19	chr13	40002361	insert	91	CACACATTAGCTCAAATGGAAG	GGTATAGTTTTGGGGCATATCA	91	91
Indel13-20	chr13	42994633	insert	129	TAATGCTTTACGTGAGAGTCTT	TGAAGACTGCCCGTACAAAGAT	0	0

Indell14-1	chr14	842506	insert	111	ATTCTTGCAGGCAAACTTTTGT	CATCACGAGAACAAGTACCAAG	0	0
Indell14-2	chr14	1089439	insert	153	GTTCATTGCGCACTTTGATATT	AATTATCCAAAAATCGGGGACA	153	153
Indell14-3	chr14	1305016	insert	107	GAGAAATGCAGAAACACAGAAG	CGATACAATAATATCGTGCTCG	144	251
Indell14-4	chr14	1605827	insert	140	AAGAGTTACGTTAGGGAAGATG	CTTGCATAAATTGAGACGGCAT	140	140
Indell14-5	chr14	2105965	insert	194	CTGGTAGGGTTCAATATTTTGA	TACTCATAGAAGGGTTTAGCCG	0	0
Indell14-6	chr14	2709835	insert	263	CACAGATGAGAATAGTTGTTTT	CAGAGATCAATGACCAATATGA	263	263
Indell14-7	chr14	3400573	insert	191	GATGTGGGTCCTTCTTAGCTAG	AGTGAATGATTGCAAGCTTAAT	191	191
**Indell14-8**	**chr14**	**5101340**	**insert**	**237**	**GCCACACAGCTCAAAGGATT**	**TCGTGGGATGTCCAAGCTGT**	**145**	**382**
Indell14-9	chr14	6962348	insert	180	CCAGGATTGTTATTTTAGTTGT	CTTAACGAATGTCTAGTCAACA	180	180
Indell14-10	chr14	8063036	insert	215	AGTGTTCTCAACAATGAGTATG	TTGATGCAAGGACCAAGTTGTT	215	215
Indell14-11	chr14	9135887	insert	171	CTACAAAGCTTCAACACAAAAG	GGAAAATTTCTCGATTGGAGAC	0	0
Indell14-12	chr14	12386477	insert	177	TCATGCTAGATATGTGAACATC	CCACAGGGACTGATAAACAACT	177	177
Indell14-13	chr14	15387559	insert	156	CTGAATAAGTTTGGTGGTTTTA	AACACACAAGGGCAAATGACAT	156	156
**Indell14-14**	**chr14**	**17402297**	**insert**	**185**	**TCTCCTCTCTCCCACCACC**	**CCTTTGGAGCTTATGGGGGA**	**221**	**406**
Indell14-15	chr14	21402309	insert	74	GCTACACATTTTGATAAGTTGA	TCGAGCTATTTTCGACTCTCTG	0	0
Indell14-16	chr14	25414940	insert	226	GAAAACTCACCAATCATGTTGG	GAGGGCCAACCATAATTTATTA	226	226
**Indell14-17**	**chr14**	**26426999**	**insert**	**233**	**TCAAAGTATAGCATACATAC**	**GTTAGCCTTATTACAAACCCA**	**176**	**409**
Indell14-18	chr14	27441667	insert	89	AGGAAAAGGTAGGGTTTGGGA	AATATGTGGGGCGAGAATAAAA	155	244
Indell14-19	chr14	29858824	insert	73	AGTAGGATGTCTGACTTATCAC	TTCGTCTAAACGGTTACCCATA	73	73
Indell14-20	chr14	31685959	insert	83	GGTGCCTCTTCGATTTTTGAGC	AAATCAAAGAGGCACCACTCTC	0	0
Indel15-1	chr15	730752	insert	87	TGGTTACGCGAATATCAGGTTT	ACTAGTAGTGTACATTTTGCAC	87	87
Indel15-2	chr15	856148	insert	124	TCGAGAACGAATGAAGAGTTTA	ATACGATCGGTACTATGATACC	124	124
Indel15-3	chr15	916931	insert	85	GCTGCAGTCAATTAACAAAAAA	ACTTTTAAAGTGGACCCCCTGT	0	0
Indel15-4	chr15	1050943	insert	119	GCAGAAGGAGATATCAGTTGCG	GCGGTTTCTCTCCTTCATAAAA	0	0
**Indel15-5**	**chr15**	**1340537**	**insert**	**115**	**AGATGAGGTTATAATTTCCTT**	**ATACTACTATAATCGTGTCCT**	**250**	**365**
Indel15-6	chr15	1789633	insert	119	CCGTCTGTTGAACTATACTACC	TAATGATGTGTTTGACGAACCC	177	296
Indel15-7	chr15	2399633	insert	136	CTCACACCTCTATTAAACTTCT	TGTGTGTGTGTGTGTGTGTGTG	136	136
**Indel15-8**	**chr15**	**2703378**	**insert**	**318**	**AATAAGAGAGCGTGCCTCGG**	**ATGAGGCCTGGAAGAGTGAT**	**135**	**453**
Indel15-9	chr15	3149489	insert	226	CACTGTATATTGTCCCTCCTAC	CCTTATTGTGTGAGATTGATCA	226	226
Indel15-10	chr15	4046664	insert	95	GGATGTCATCCCCTTCTAAAGG	AAACCCTACTAGCTAAAACCCT	0	0
Indel15-11	chr15	4989318	insert	92	AGTCGGTTGCATGAAATTTCTA	AAACTAGGTCCTCGAGGTAGCT	92	92
**Indel15-12**	**chr15**	**6265907**	**insert**	**285**	**GGTTTAATCATTTATTTATGGC**	**TATTTGGCTATAAAGTCCTTT**	**140**	**425**
Indel15-13	chr15	9530642	insert	133	ATAAATAACAGCAGTTACCCAC	GCACTTTTAATCACTGTTTACT	133	133

Indel15-14	chr15	11655244	insert	97	AGGTCTTATTAGGCTTGATAAG	TGTTTAGATCCTGATAGGGTAG		
Indel15-15	chr15	13883973	insert	81	TTATTTCCCTCCCCATTTCCCG	AAAACCGGCCAAACTTAACCCT	190	271
Indel15-16	chr15	18103905	insert	173	GCAAGAATACAAAGATGTTGTA	ATGGTTTTTCAAAGGATTTTCA		
Indel15-17	chr15	20029341	insert	85	AACAGGTTTTCTAAAAACTAGG	AAGCATCTGATCGTGACGAATT	0	0
Indel15-18	chr15	21374302	insert	155	TTTGACGTAGGTAATTAGAGCA	AATCTAGTTGATCTAGTTGAGG	155	155
Indel15-19	chr15	23589371	insert	212	CCCTTTACATGAGTTCAAAACT	GGAAGGACTTTAACTCGAGATT		
Indel15-20	chr15	25825175	insert	173	GTTTAATTACAAGCACCAGAGA	TAGCATTGTGATTGTCGGACTC	110	283
Indel16-1	chr16	850778	insert	106	CGCAAGGAAAAAGAGAAAATGT	CCACCGTCTATAGTTAAAACTT	0	0
Indel16-2	chr16	923722	insert	104	TCGTCAAGAAATTAGAGAGAAG	ATTTTAAAGAAACGTAAAAGCC	104	104
Indel16-3	chr16	1204640	insert	88	CTTGATGCTGTGTGAGAAACTT	CTGACCTTCCAATTCAAAAGTT	0	0
Indel16-4	chr16	1700594	insert	139	AGCATTCATTAGATTGCTTGTC	CAGAGTAGTGTTCAATTTGGCG	139	139
**Indel16-5**	**chr16**	**2860104**	**insert**	**217**	**ATGTGAGAGTATTCAACGCA**	**CCCACTTCAATCTAGTGCTAA**	**150**	**367**
Indel16-6	chr16	3127043	insert	107	CCTTTTGATAAAGTTTCAACCT	ATTTGGCCATGAAAGGTCTTG	107	107
Indel16-7	chr16	4152210	delete	97	TTTAACATAGGGGACCTGAAGC	AAATCTTCTTCTCTTTGACCAA	0	0
Indel16-8	chr16	4765376	insert	108	AAACTCCTGAAGGCTTGCGCCT	GGTTTTCTTGCAGCTTATTCGG	108	108
Indel16-9	chr16	4989870	insert	85	GTTCTATGGCCTTTGGAGACCA	GTTTGGGCCTAAAGTTGTTGTC	187	272
Indel16-10	chr16	5100893	insert	97	ACAGAAAGGAGCAGGGATGTAT	CCGGAGTCTATTATTGTTCACT	0	0
Indel16-11	chr16	5485902	insert	145	TACTGTATGCATGTGGGCATAC	TCGCTAAATCACTGTGGTTTG	145	145
**Indel16-12**	**chr16**	**5730852**	**insert**	**197**	**ATTGGAATCTAAAATAGTTTC**	**TGGTTCAAATCGGACTGTAA**	**125**	**322**
Indel16-13	chr16	6090989	insert	301	CTCTGATGTGAGGTGGATGATG	TCGGGGGCATTCTCACCATATT	301	301
**Indel16-14**	**chr16**	**6832870**	**insert**	**283**	**TGGTAAGGTTTGGTTTGAAGC**	**CATAATTATCCGATCCCACTT**	**133**	**416**
Indel16-15	chr16	9085938	insert	92	CTTTCTTTCATTTTCCTTGTTT	CTCTTTGACCGAAAAGAACGCG	92	92
Indel16-16	chr16	12222319	insert	97	CATAGGGTTTTGGTTGACAATA	GGAAAATAATGAGGTCAGGGAT	0	0
Indel16-17	chr16	22206219	insert	111	GTCGATGTTAAGATGAATAGCA	TCGAGTTCGATGCTCTTCTTTC	120	231
Indel16-18	chr16	26543135	insert	91	GTAGATAACCTTCATCGGCCGC	CATTTCACCCTCAGGACAAAGA	91	91
Indel16-19	chr16	28068257	insert	189	GGCCGGAGATAACATGAATCTC	TTCTGACCTGATTGCGCATCTC	189	189
Indel16-20	chr16	30201389	insert	198	GAGAGACAAGAAAGCTCTCTAC	TTTAGTTTCTTCAATCGTCGCG	0	0
Indel17-1	chr17	734316	insert	112	GAGTCTTTCCGTCTGTCTTCTA	CTGTCTGAGGCAACTTGATCTG	112	112
Indel17-2	chr17	958819	insert	107	AGCTGGTTTCGCTGTACATAAA	TTAAACCTAGAACTCCGCACTG	107	107
Indel17-3	chr17	1008196	insert	90	CCTGTTCTCCTATTAACAAAAA	CATCAAAAGCTTGCTAAAACAC	0	0
Indel17-4	chr17	1453386	delete	119	AGAGCGAAAATTTTGCAGCGTG	TAAATTGACCGTACGCTCTCCT	0	0
Indel17-5	chr17	1622726	delete	113	ATGTAAAACTAAAAGCCAACGG	ATCTTTCTCGGCATGTTAATAC	332	219
Indel17-6	chr17	1999217	insert	85	GATACCAGAAGTTTGAAAAATG	TACCTGTTGTCCATGCATGACG	85	85

Indel17-7	chr17	2450065	insert	89	ATCTTGCCTGCGATGATGATTG	CTTTTGTTGTCGTTTTCGAATC	0	0
Indel17-8	chr17	2877043	insert	175	AGCCTCTTGGGAAACCCCTTTC	GGTTCGATAAAGGGACTTGAGA	175	175
Indel17-9	chr17	3466239	insert	256	ACGAATGTTGATAATGTCAATG	TTGTGGCCATTTATTTTTTCCA	256	256
**Indel17-10**	**chr17**	**6473938**	**insert**	**287**	**TTCTGCCACCACGAGAGC**	**ATGTGGCTCGTCTCTGCG**	**152**	**439**
Indel17-11	chr17	8794135	insert	85	AGATTTGTTGAAGACAAAAATC	GATGCCAAGTTGGCGGATACAG	0	0
**Indel17-12**	**chr17**	**10095497**	**insert**	**211**	**TGTGGAATCATCATTTTACCT**	**CGGGTATCATGGTGAAAAATT**	**167**	**378**
Indel17-13	chr17	11069737	insert	103	TAGGCAGTAGAGAAGTGGTCTG	CCAGACGATTCGATACCCGAAC		
**Indel17-14**	**chr17**	**16278884**	**insert**	**190**	**ACCAAAATACCGTCAGAACA**	**CTCTCTCAAACGTCATGTGATT**	**205**	**395**
Indel17-15	chr17	21022397	insert	80	ACCGGCAATGTAAGATAAACTT	GTATACACGGAGACAGGGAGGT	80	80
Indel17-16	chr17	27326829	insert	109	ACATCCAACGAAGCATCCTCAT	CGTTGGTAATGGATGAGTTAAC	0	0
Indel17-17	chr17	29137245	insert	86	GATATCATTCGCCGCTACCTAA	TCCGTCTGCATGACATTTAGAA	141	227
Indel17-18	chr17	31575111	insert	106	GATCGACTATGTTGTTTCTGCT	GCCCCTTGATTCAAATCGAACCA	0	0
Indel17-19	chr17	32099352	insert	131	CGTAACACCATCCTTAGAAATC	TTTTGCAAAACAAGACTAGTCT	131	131
Indel17-20	chr17	32960171	insert	98	TATGTGGAAAGTTGCTTCTTGG	GTGAGAAACCAAAAGCAAAAGA	137	235

Table S2 Information on Indel markers that are invalid in three hybrid populations.

Population	Invalid Indel
'Golden Delicious' × 'Jin Zhui'	Indel1-13，Indel4-14，Indel6-13，Indel4-8，Indel12-17,Indel15-8
'Fuji' × 'Yan Zhuang'	Indel1-13，Indel2-16，Indel5-5，Indel6-10，Indel7-6，Indel8-10，Indel8-16，Indel10-4，Indel11-12，Indel13-7，Indel15-12，Indel16-5
'Fuji' × 'Jin Zhui'	Indel1-13，Indel5-5，Indel6-10，Indel6-13，Indel7-18，Indel8-10，Indel8-19，Indel9-10，Indel10-17，Indel11-12，Indel12-6，Indel13-7，Indel14-17，Indel15-12，Indel16-5
